# Replica Exchange Molecular Dynamics with Hydrogen Mass Repartitioning Improves Sampling of Peptide Binding to a Lipid Bilayer

**DOI:** 10.1021/acs.jctc.6c01064

**Published:** 2026-08-04

**Authors:** Steven R. Bowers, Christopher Lockhart, Dmitri K. Klimov

**Affiliations:** School of Systems Biology, 114334 George Mason University, Manassas, Virginia 20110, United States

## Abstract

We compared the performance of two mass repartitioning models, HMR3 and HMR2, with tripled and doubled hydrogen masses, against the model with standard masses (SM). Due to heavier hydrogens, HMR3 and HMR2 afford longer integration steps of 4 and 3.5 fs, respectively, whereas SM used a 1 fs time step as a reference. All-atom replica exchange molecular dynamics simulations of the antimicrobial peptide PGLa binding to an anionic DMPC/DMPG bilayer were used as a case study. Across all conditions, HMR3 and HMR2 simulations each collected 36 μs of sampling, which is 50% more than our previous SM simulations. Therefore, the motivation for our work was to evaluate the HMR performance in complex biomolecular systems coupled with an advanced sampling algorithm. Our investigations led to three main conclusions. First, by affording longer simulations, HMR3 and HMR2 models provide better equilibration of PGLa peptide binding to the lipid bilayer. Specifically, they eliminated the metastable surface-bound (SB) state observed in SM. In the mass repartitioned models, PGLa exclusively sampled the inserted state (I), which also appeared as a dominant state in SM but alongside the SB state. Importantly, this better equilibration of peptide binding improved the consistency with experimental data. Second, HMR3 and HMR2 closely reproduce a broad range of structural properties previously reported for SM after correcting them for equilibration. Additionally, HMR3 and HMR2 demonstrate excellent agreement between themselves in sampling the conformational ensemble. These findings indicate that the mass repartitioning models preserve structural properties in this complex biomolecular system. Third, the actual acceleration of sampling by HMR3 is about 3.5-fold, which is lower than the theoretical 4-fold gain. This slight underperformance may be due to the slow dynamics of heavy hydrogens. HMR2 shows qualitatively similar results. An increase in the integration step in SM to 2 fs or adjustments in the computations of long-range interactions may reduce the computational gains offered by mass repartitioning, but do not negate their advantages in efficiency. HMR2, and particularly HMR3, are excellent options for accelerating conformational sampling in complex biomolecular systems.

## Graphical abstract

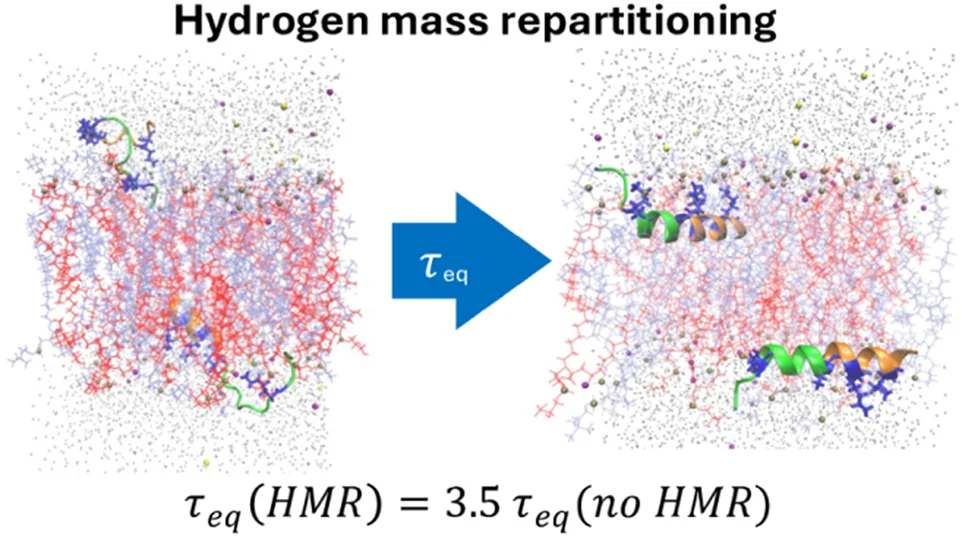

## 1 Introduction

All-atom molecular dynamics (MD) simulations provide a rigorous “physics-based” description of biomolecular systems. Their most valuable advantage is the ability to transgress scales, from microscopic atomistic properties to macroscopic observables. One of the main problems plaguing MD simulations lies in the difficulty of achieving convergent sampling of states consistent with the underlying free energy landscape. They are caused by the ruggedness of free energy landscapes, which, under normal physiological conditions, feature deep basins separated by high (compared to RT) barriers. The convergence problem can be partially addressed by enhanced sampling algorithms, particularly by replica exchange molecular dynamics^1–4^ and its variants, replica exchange with solute tempering and replica exchange with hybrid tempering (abbreviated as REST and REHT, respectively).^5,6^ Over the years, these simulations were successful in probing the thermodynamics of various biomolecular systems.^7–17^ Nevertheless, converging REMD simulations typically extend into the microsecond scale, translating into a severe computational burden.^18^ An approach complementary to enhanced sampling relies on increasing the length of the integration step δ*t* beyond the typical values of 1 or 2 fs. An immediate obstacle to a straightforward increase in δ*t* arises from the instability of the SHAKE algorithm, which is commonly used to constrain the lengths of hydrogen-related covalent bonds.^19^ This problem can be solved by making hydrogen atoms heavier, which would damp their high-frequency motions and allow one to maintain long δ*t*. Although changing hydrogen masses results in artificial biomolecular systems, they retain the thermodynamic properties of those with standard masses due to the separation of kinetic and potential energy terms in the configurational integral. Consequently, the canonical distribution of states stays independent of atomic masses.

To prevent an increase in system viscosity due to heavier molecules, a proper implementation of heavy hydrogens warrants mass repartitioning that balances the increase in hydrogen mass with a proportionate decrease in the masses of heavy atoms. This approach, referred to as hydrogen mass repartitioning (HMR), was thoroughly investigated by Berendsen and coworkers and, more recently, by Roitberg and coworkers.^19,20^ Those studies showed that HMR MD simulations remain stable up to the nanosecond time scale with the integration step δ*t* = 4 fs. HMR has also been applied to accelerate the MD simulations of lipid bilayers with embedded proteins.^21^ The authors found that the HMR model with δ*t* = 4 fs does not change the properties of pure DPPC lipid bilayers, such as area-per-lipid, electron density profiles, or *S*_CD_ order parameters, as expected. Even if peptides or proteins are inserted in the bilayer, HMR does not skew the conductance through the membrane, hydrogen-bond distributions, or mixing of POPC and cholesterol. More recently, our group has investigated the coupling of REHT simulations with HMR by probing the thermodynamics of alanine dipeptide.^22^ We found that HMR preserves the peptide free energy landscape together with its structural properties, including hydrogen bonding, water density in the first solvation shells, and RMSD distributions. Importantly, we showed that HMR boosts conformational sampling by more than 3-fold with the adjustment of the computation of long-range nonbonded interactions.

However, it remains unclear if HMR models can be successfully and efficiently used in challenging REHT simulations probing the thermodynamics of peptide-bilayer interactions. More specifically, do HMR models meaningfully accelerate and preserve the mechanism of binding of the antimicrobial peptide PGLa to anionic lipid bilayers? This 21-residue cationic peptide is an excellent test case because it has been extensively studied experimentally and computationally.^23^ For example, circular dichroism data indicated that PGLa in lipid-free water is unstructured,^24^ but binding to DPC micelles converts its random coil state into a helical state.^25^ There is a considerable body of experimental information concerning the PGLa position in lipid bilayers. Solid-state NMR experiments have determined that when the bilayer exists in the liquid crystalline phase, PGLa adopts a surface-bound S-state, provided that the peptide:lipid ratio is low (1:200).^26^ In this state, PGLa is positioned almost parallel to the DMPC/DMPG bilayer surface with an upward orientation of lysine side chains.^27,28^ However, at higher peptide:lipid ratios, PGLa may transition to a tilted T-state.^27–30^ Moreover, the PGLa states are affected by the thickness of the bilayer hydrophobic core,^29^ temperature, and bilayer phase.^31^ The experiments also revealed that PGLa binding causes disorder in the structure of DMPC and DMPG lipids.^32^

To study the mechanisms of equilibrium PGLa binding to anionic DMPC/DMPG bilayers, we have previously used various replica exchange algorithms.^33,34^ In the limit of a low peptide:lipid ratio (1:200), we computed the binding free energy landscape and found two major PGLa-bound states: a metastable surface-bound (**SB**) state and a dominant inserted (**I**) state. In both states, the peptide adopts a stable helix conformation. The positively charged amino acids in PGLa formed strong electrostatic contacts with anionic phosphate groups, forcing the peptide to rotate as it transitions from the **SB** to **I** states and maintains these contacts. Interestingly, PGLa binding induces an influx of anionic DMPG and an efflux of zwitterionic DMPC lipids into and from the peptide binding footprint. We concluded that the major factors governing PGLa binding to the DMPC/DMPG bilayer are the desolvation of PGLa positive charges and the formation of electrostatic PGLa–lipid interactions.

Although our previous simulations described the PGLa binding in broad agreement with experimental observations, they left lingering questions. One of them is the extent of full equilibration of the bound PGLa peptide within the bilayer, which may impact the weights of **SB** and **I** states. We previously found that an application of the REHT algorithm vastly improved the equilibration of PGLa in the bilayer compared to REST simulations.^34^ However, even 1.2 μs of REHT sampling at 310 K could not unambiguously rule out the transient nature of **SB**, which is not observed experimentally. An additional reason for the current work is further testing of HMR. We have shown that HMR simulations significantly accelerate sampling for an alanine dipeptide,^22^ but whether they hold the same advantage for a complex biomolecular system is unclear. To address these concerns and to test the utility of HMR, we coupled this model with REHT simulations to examine PGLa binding to the DMPC/DMPG bilayer. We increased the amount of sampling by 50% to almost 2 μs at 310 K. To verify that our HMR sampling is unbiased, we tested two mass repartitioning schemes in which the mass of hydrogen is doubled and tripled. We show that further equilibration of the bound PGLa eliminates the metastable **SB** state. We also demonstrate that as long as a structural quantity is not affected by **SB**, it is well preserved across the models with and without mass repartitioning. Perhaps more importantly, we show that HMR reduces the computational effort compared to REHT simulations with unchanged hydrogen masses, although the actual gains may depend on the structural quantities and simulation details.

## 2 Models and Methods

### 2.1 All-Atom Simulation Model

To test the application of hydrogen mass repartitioning (HMR), we selected the same model already used by us to study PGLa peptide binding to an anionic lipid bilayer^33,34^ (Figure 1). The simulation system contained a DMPC/DMPG (3:2) lipid bilayer composed of 60 zwitterionic DMPC and 38 anionic DMPG lipids, two PGLa peptides binding to opposite bilayer leaflets, 4410 TIP3P water molecules, 38 sodium and 10 chlorine counterions. The ions set the system’s net charge at zero and introduce salt. Because the initial system dimensions were 55 Å × 55 Å × 82 Å, the effective salt concentration is ∼70 mM or ∼110 mM if we consider only the volume available for water. The peptide N-terminus was protonated and thus positively charged, while the C-terminus was amidated.^23^ Therefore, the net peptide charge was +5. Following our previous simulations, we chose the all-atom CHARMM36 force field^35^ for lipids and the all-atom CHARMM22 force field with CMAP corrections^36^ for the peptides. The rationale for using this parametrization is discussed in the Supporting Information (SI). The DMPC/DMPG bilayer approximately emulates an anionic bacterial membrane and has been well studied experimentally.^37^ The symmetric design of the simulation system is computationally efficient, allowing us to double conformational sampling and reduce the pressure disparity across the leaflets.^38^

**Figure 1:**
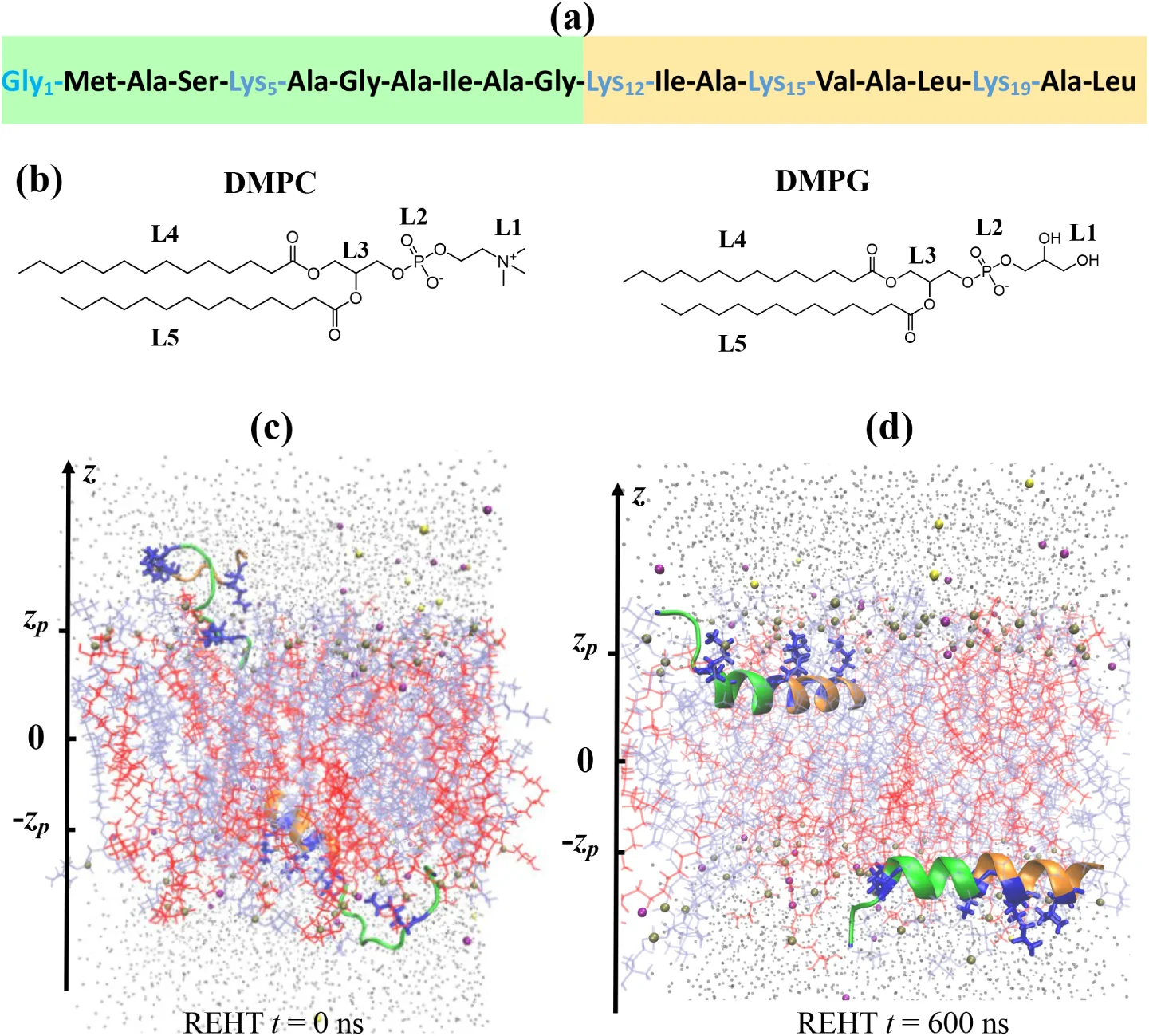
(a) PGLa peptide sequence with positively charged amino acids in blue. N- and C-termini are in green and orange, respectively. (b) The chemical structure of DMPC and DMPG lipids. Lipid structural groups are marked. (c, d) The conformational snapshots of the HMR3 simulation system at the start and end of the REHT trajectory at 310 K. Two PGLa peptides colored following panel (a) bind the opposite leaflets of the bilayer. Lysine amino acids use licorice representation. The DMPC and DMPG lipids are in pale blue and red, respectively. Their phosphorus atoms are in tan. Gray dots above and below the bilayer are water molecules, while sodium and chlorine ions are purple and yellow spheres.

We used two HMR models. Most of our analysis has been performed for HMR3 simulations, in which the mass of all hydrogens in the system is tripled to *m*_H_ = 3.0240 amu (Figure 1c,d). To assess the impact of hydrogen mass, we repeated the simulations using HMR2 with doubled hydrogen mass (*m*_H_ = 2.0160 amu). To keep the total masses of molecules unchanged, the masses of the heavy atoms bonded to hydrogens were proportionally reduced. Whenever possible, we compare our findings to previous simulations with standard masses (SM).^34^ It must be noted that executing HMR simulations with NAMD requires care.^22^ NAMD 3.0.2 does not apply SETTLE restraints to repartitioned water, forcing HMR2 and HMR3 simulations to utilize SHAKE for all hydrogens, which makes water flexible. As a result, the HMR energy function as related to water differs from the standard mass model. However, our previous study showed no distinction between water–peptide interactions in flexible and rigid water models.^22^ Nevertheless, if water remains rigid due to SETTLE restraints, HMR should not include water, as its application would only increase water viscosity.

### 2.2 Conformational Sampling

This study combines replica exchange with hybrid tempering (REHT)^6^ enhanced sampling with HMR to investigate the binding of PGLa peptides to the lipid bilayer. The REHT implementation follows our previous studies.^22,34^ Briefly, REHT replicates the molecular system *R* times, subjecting each replica to a unique condition *m* (0 ≤ *m* ≤ *R*–1). These conditions are determined by the two temperatures *T*_*m*_ and $T_{m}^{\prime }$ , which define the enthalpy as

1 $$H_{m}=E_{p}+\sqrt{\frac{\beta_{m}^{\prime }}{\beta_{m}}}E_{ps}+\frac{\beta_{m}^{\prime }}{\beta_{m}}E_{s}+\mathrm{PV}$$

where *E*_p_, *E*_s_, and *E*_ps_ are the energies of solute (p), solvent (s), and solute–solvent interactions, β_m_ = 1/(*R*_c_ *T*_m_), and *R*_c_ is a gas constant. For *m* > 0, the temperatures $T_{m}^{\prime }<T_{m}$ , whereas $T_{0}^{\prime }=T_{0}$ . Equation 1 shows that REHT fully tempers solute with the temperatures *T*_*m*_ but applies limited tempering to the solvent by scaling up its interactions. Because the effective temperature of the solvent is $T_{m}^{\prime }$ , its conformational space is “colder” than that of the solute at *m* > 0. Compared to the original replica exchange method,^3^ limited solvent tempering reduces the number of conditions *R*, which still span a temperature range with adequate exchange rates. In our REHT simulations, the solute includes both peptides and counterions, whereas the solvent consists of lipids, water, and remaining ions. The Metropolis criterion swaps the replicas *r* and *r* + 1 at adjacent temperatures *T*_m_ and *T*_m+1_ with the probability ω = min­(1, *e* ^–Δ^), where Δ = β_*m*_(*H*_*m*_(*x*_*r*+1_)-*H*_*m*_(*x*_*r*_))+β_*m*+1_(*H*_*m*+1_(*x*_*r*_)-*H*_*m*+1_(*x*_*r*+1_)), *x*_*r*_ and *x*_*r*+1_ are the coordinates of replicas *r* and *r* + 1. These replica swaps results in a random walk of replicas over conditions *m*. Although solvent is effectively colder than solute at *m* > 0, REHT rigorously maintains equilibrium.

Following our previous study,^22^ we scaled the integration steps δ*t* to stabilize HMR models at elevated REHT temperatures. Specifically, the average atomic displacements at the lowest *T*_0_ and given REHT *T*_*m*_ temperatures were set to be the same, i.e., δ*t*(*T*_0_)*v*(*T*_0_) = δ*t*(*T*_*m*_)*v*(*T*_*m*_), where *v*(*T*) is the average thermal velocity at temperature *T*. From the Maxwell–Boltzmann distribution, we find that $\delta t\left(T_{m}\right)=\left[v\left(T_{0}\right)/v\left(T_{m}\right)\right]\delta t\left(T_{0}\right)=\sqrt{T_{0}/T_{m}}\delta t\left(T_{0}\right)$ . As shown in previous studies, HMR2 and HMR3 models afford stable simulations with δ*t* = 3.5 and 4.0 fs at *T*_0_ = 310 K.^19–22^

REHT simulations were performed using NAMD and in-house scripts managing replica exchanges.^39^ We used *R* = 20 conditions $\left(T_{m},T_{m}^{\prime }\right)$ , in which the temperatures *T*_*m*_ were distributed exponentially in the range from *T*_0_ = 310 to *T*_*R*‑1_ = 430 K. The effective solvent temperatures $T_{m}^{\prime }$ were exponentially distributed from $T_{0}^{\prime }=310$ to $T_{R-1}^{\prime }=350K$ . All conditions $\left(T_{m},T_{m}^{\prime }\right)$ are listed in Table S1 in Supporting Information. Replica exchanges were attempted every 2 ps at 310 K, independent of the chosen δ*t*. However, due to δ*t* scaling, the interval between exchange attempts at *m* > 0 is shorter. REHT simulations used isobaric–isothermal (NPT) ensembles, in which pressure was kept at 1 atm using the Nosé-Hoover Langevin piston method. Compared to SM simulations^34^ the piston period and decay were increased to 400 and 200 fs, respectively. Temperature was maintained by an underdamped Langevin thermostat with a damping coefficient γ = 5 ps^–1^. Presence of the bilayer necessitated a semi-isotropic pressure scheme that coupled the *x*,*y* dimensions leaving *z* to adjust independently. All simulations used periodic boundary conditions. Electrostatic interactions were computed via Ewald summations, whereas van der Waals interactions were smoothly switched off using force switching in the interval from 8 to 12 Å. Independent of δ*t* the Verlet lists were updated every 20th integration step, while long-range electrostatic interactions were evaluated every second step.

To facilitate the comparison across HMR and SM models, HMR simulations used the same initial conditions as SM.^34^ Compared to SM simulations, which sampled 400 ns per trajectory and replica, the new HMR3 and HMR2 simulations reached 600 ns. In all, we produced three REHT trajectories for each HMR model. Therefore, the total amount of sampling at 310 K was 1.8 μs, whereas the sampling across all conditions reached 36 μs. Convergence analysis (see below) suggests that REHT simulations at 310 K approximately equilibrated after *t*_eq_ = 500 ns. If so, the amount of equilibrium sampling at 310 K is 300 ns per HMR model.

### 2.3 Computation of Structural Probes

The following structural probes describe PGLa binding to the bilayer. Peptide secondary structure was analyzed using STRIDE.^40^ The helical propensity *H*(*i*) for amino acid *i* is given by the probability of observing α-, 3_10_-, or π-helices at *i*. An amino acid and lipid group (Figure 1b) form a contact if their centers of mass are less than 6.5 Å apart. PGLa is bound to the bilayer if it forms at least one such contact. To probe peptide hydration, we assume that a water molecule is in the first solvation shell of an amino acid if its oxygen atom is within 4.5 Å of any amino acid heavy atom. The hydration is then quantified by the number of water molecules in the first solvation shell ⟨*N*_w_⟩.

The insertion of amino acids into the bilayer is tracked with the probabilities *P*(*z*;*i*) for the amino acid *i* side chain center of mass to occur at *z*. If an amino acid center of mass *z*(*i*) is below *z*_*P*_, an amino acid is inserted. If *z*_*P*_ < *z*(*i*) < *z*_*P*_ + 6.5 Å, an amino acid is surface-bound, and when *z*(*i*) > *z*_*P*_ + 6.5 Å, it is unbound. To probe lipid distributions in the bilayer, we computed the volume number densities of lipid heavy atoms *n*_*v,l*_(*r*,*z*) as a function of the distance *r* to the peptide center of mass in the (*x*, *y*) plane and the distance *z* to the bilayer midplane. The density *n*_v,l_(*r*,*z*) reports the distribution of all lipids and is computed by permuting the upper and lower leaflets and placing the PGLa center of mass at *r* = 0. The number density *n*_w_(*r*,*z*) of water oxygen atoms mapped the water distribution. The lipid–water boundary *z*_b_(*r*) in the distant (from the peptide) region follows from the condition *n*_w_(*r*,*z*_b_(*r*)) = *n*_v,l_(*r*,*z*_b_(*r*)). We assumed that in this region *z*_b_(*r*) = *z*_0_ does not depend on *r*. With this definition, the distant region occurs at *r* > *r*_d_ = 21.5 Å. The near bilayer region lies within $r<\frac{1}{2}\left\langle R_{g}\right\rangle =6$ Å, where ⟨*R*_g_⟩ is the peptide average radius of gyration. At *r* < *r*_d_, the bilayer boundary *z*_b_(*r*) follows from the condition *n*_v,l_(*r*,*z*_b_(*r*)) = *n*_v,l_(*r*_d_,*z*_0_). Therefore, the bilayer boundary tracks lipid density. The bilayer thickness *D* is the distance between its boundaries in the opposite leaflets. The surface lipid number densities *n*_s,*x*_, where *x* = DMPC, DMPG, or *x* = l for any lipid, were computed using the positions of lipid phosphorus atoms.

To provide direct comparison with experimental data, we computed two angles. Because PGLa predominantly adopts a helical state in the bilayer, we measure helix tilt with the angle γ. Following Reischer et al.,^41^ we divided the peptide into N- and C-terminal sections represented by the centers of mass of amino acids 6 and 14. The vector *h⃗* connecting these centers of mass defines the helix axis. The angle between *h⃗* and the bilayer normal is γ. If γ = 90°, the helix lies parallel to the bilayer surface. The rotation angle β­(*i*) reports the orientation of residue *i* in a helix state.^41^ To determine β, the PGLa helix was first rotated, aligning *h⃗* stepwith the *x*-axis. The angle between the *y*-axis and the vector drawn between the helix axis and the C_α_ atom of amino acid *i* is β­(*i*). If β = 90° or 270°, amino acid *i* is pointed up or down along the bilayer normal. The rotation angle was computed for *i* = Lys12 and Lys19.

To compute the equilibration time scales, we considered the time dependencies, *H*(*t*) and *z*_CM_(*t*), which track the helical fraction in PGLa and the position of its center of mass at 310 K. These dependencies were fit with the exponential function *a*_0_ + *a*_1_exp­(−*t*/τ), where *a*_0_, *a*_1_, and τ are fitting parameters. The latter is considered the equilibration time scale. The computational effort required to equilibrate the system was quantified by the time τ_σ_ = τ/σ scaled down by the integration step-up factor σ = δ*t*/δ*t*(SM), where δ*t* is the integration step and δ*t*(SM) = 1 fs is used in SM simulations. It is possible that, due to computational overheads, σ overestimates the actual computational gains.^42^ Using NAMD 3.02, we averaged the wall-clock time required to perform a 2 ns REHT simulation using HMR or SM models. We found that the HMR3 model with δ*t*_*s*_ = 4 fs executes these simulations 3.9 times faster than SM. HMR2 with δ*t*_*s*_ = 3.5 fs accelerates the simulations 3.4 times. Thus, σ approximates the theoretical gains in computational efficiency.

The angular brackets ⟨···⟩ indicate averages over the entire equilibrated data set computed using weighted histogram analysis.^43^ All quantities are reported at 310 K. Standard errors are computed by treating each REHT trajectory as an independent sample.

## 3 Results and Discussion

### 3.1 Hydrogen Mass Repartitioning Facilitates Equilibration

One of the key questions in our study addresses the ability of HMR models to extend sampling and promote equilibration in the complex biomolecular system. Previously, using REHT simulations without mass repartitioning (SM), we showed that binding of the PGLa monomer to the DMPC/DMPG bilayer triggers acquisition of helical structure in the peptide and leads to its insertion into the bilayer core.^34^ The length of the SM simulations was 400 ns per trajectory at 310 K, and they were deemed equilibrated after *t*_eq_ = 320 ns. In the current study, HMR3 and HMR2 simulations reached 600 ns per trajectory, starting with the same initial conditions as SM. Thus, HMR simulations produced 50% longer sampling than SM. To directly compare HMR models, we computed the helical fraction *H*(*t*) and the position of the center of mass *z*_CM_(*t*) along the bilayer normal for PGLa peptides as a function of REHT sampling time *t*. Figure 2 shows these time dependencies together with their exponential fits. The fitting parameters are listed in Table 1. Both HMR models show excellent agreement between their *H*(*t*) profiles, which reach the same baseline of ≈0.63 after *t*_eq_ = 500 ns, suggesting approximate equilibration. HMR2 and HMR3 simulations exhibit an apparent delay in helix formation compared to SM, even though all simulations shared initial conditions. Further examination of *H*(*t*) at the simulation initiation revealed a rapid partial disruption of helical structure in HMR models, which recovers with time. We determined that the initial helix disruption was caused by initializing HMR simulations with the velocities saved from SM simulations. The magnitude of SM velocities is larger than expected in HMR, causing disruption of the peptide structure (see SI for further details).

**Table 1:** Equilibration Parameters for HMR and SM Models

process	model	*a*_0_	*a*_1_	τ^a^, ns	τ_σ_, ns	$\tau_{\sigma ,cor}^{a}$ ,^b^ns
Helix formation	SM	0.78 ± 0.09	-0.42 ± 0.19	308 ± 81	308	
	HMR2	0.72 ± 0.06	-0.41 ± 0.03	393 ± 96	112	134
	HMR3	0.72 ± 0.05	-0.42 ± 0.03	352 ± 78	88	106
Insertion	SM	11.3 ± 1.3	9.9 ± 1.4	171 ± 51	171	
	HMR2	10.7 ± 0.8	9.7 ± 0.6	166 ± 15	47	57
	HMR3	10.7 ± 0.5	9.3 ± 0.4	192 ± 43	48	58

a τ reflects the equilibration time scales extracted from the exponential fits. They should be distinguished from *t*_eq_ measuring the equilibration to an approximate baseline. As such, *t*_eq_ is approximate and may depend on the simulation length.

b The time scales *τ*_*σ,*cor_ reflect the adjustments in computing long-range interactions.

**Figure 2:**
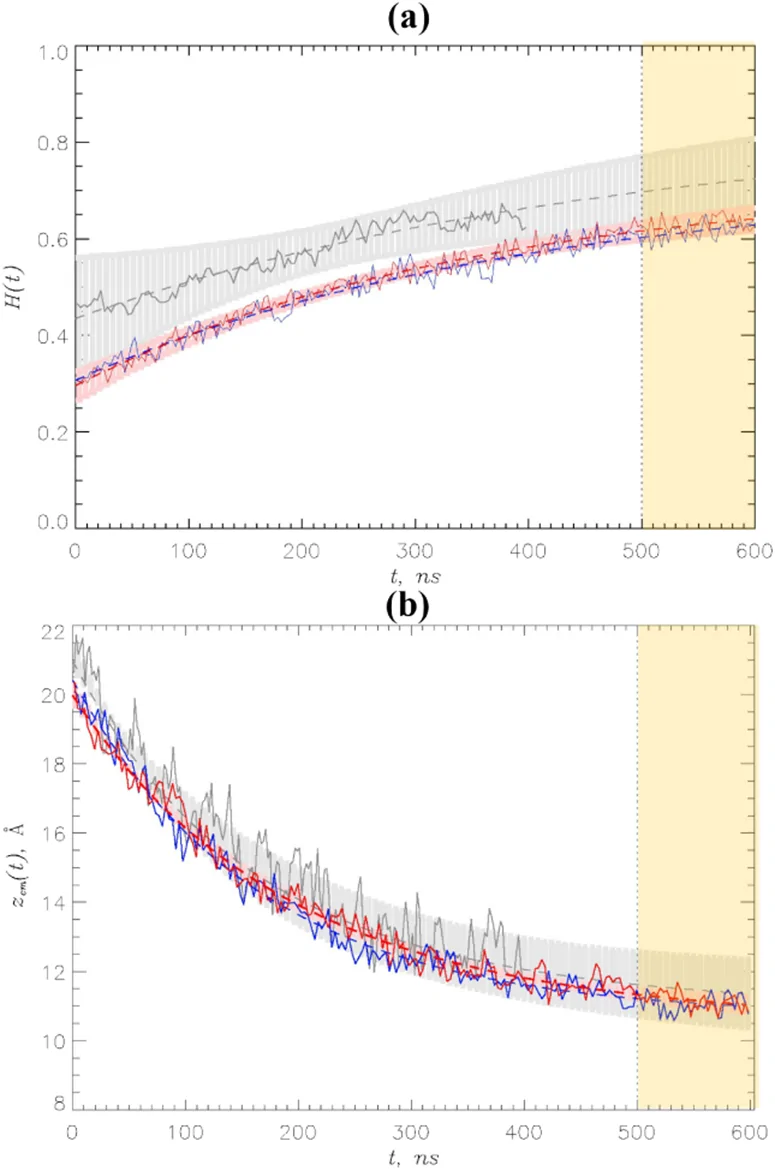
PGLa helical fraction *H*(*t*) (a) and the position of its center of mass *z*_CM_(*t*) along the bilayer normal (b) are plotted as a function of REHT sampling time *t*. In both panels, the data in red, blue, and gray refer to HMR3, HMR2, and SM^34^ models, respectively. The shaded region at *t* > *t*_eq_ = 500 ns corresponds to the apparent equilibrium sampling in HMR2 and HMR3. Dashed lines represent the exponential fits, whose fitting parameters are given in Table 1. The SM and HMR3 fitting errors are shown by respective pale color bands. Apart from the delay in helix formation, the three models with (HMR2 and HMR3) and without (SM) mass repartitioning display good agreement within the sampling errors.

Figure 2b compares the insertion progress between HMR3 and HMR2 models, revealing a near-perfect match between their *z*_CM_(*t*). Compared to SM, HMR3 and HMR2 simulations exhibit deeper insertion of PGLa into the bilayer. An apparent equilibration of peptide position in HMR models occurs at *t* > *t*_eq_ = 500 ns, where *z*_CM_(*t*) approaches the baseline ≈11 Å. Because, in this time interval, the helical fraction *H*(*t*) also shows signs of leveling off, we assume that *t*_eq_ corresponds to the onset of approximate equilibrium in HMR3 and HMR2 simulations. Taken together, Figure 2 leads to three conclusions. First, HMR models uncover a longer than previously assumed equilibration process in PGLa binding to the DMPC/DMPG bilayer. If measured by the approximate *H*(*t*) and *z*_CM_(*t*) baselines, the equilibration time *t*_eq_ is at least 500 ns, which is longer than the older estimate of 320 ns. Consequently, all HMR equilibrium properties below are reported for *t* > *t*_eq_ = 500 ns. In this context, we draw a distinction between equilibrium time scales τ and the time *t*_eq_, as indicated in the footnote to Table 1. Furthermore, one needs to differentiate fitting parameters *a*_0_ in Table 1 and equilibrium properties computed at *t* > *t*_eq_. The former predict the asymptotic equilibrium values, whereas the latter are their approximations based on the operational definition of equilibrium. Second, the fitting parameters *a*_0_ in Table 1 allow us to compare the apparent thermodynamic averages for the insertion depth and helical fractions. Because they are consistent within the error estimates across all models, mass repartitioning does not change the thermodynamic properties (see also below). Third, an excellent agreement between HMR2 and HMR3 supports the accuracy of mass-repartitioned simulations.

### 3.2 Structure and Position of the PGLa Peptide in the DMPC/DMPG Bilayer

One of the hallmarks of PGLa binding to the lipid bilayer is the appearance of a helical structure. We computed the PGLa secondary structure sampled in HMR3 simulations and found that the peptide equilibrium helical propensity reaches ⟨*H*⟩ = 0.64 ± 0.02. The C-terminus Ct exhibits a stronger helical propensity of 0.77 ± 0.01 than the N-terminus Nt, in which it is reduced to 0.51 ± 0.04. Further details are provided by Figure 3, which shows the appearance of a stable helix ⟨*H*(*i*)⟩ > 0.5 at the amino acid positions 5 ≤ *i* ≤ 21. Within the sampling error, the HMR2 data in Figure 3 match those of HMR3. Indeed, the overall helical propensity of PGLa in HMR2 simulations is ⟨*H*⟩ = 0.62 ± 0.02, with those in the N- and C-termini being 0.48 ± 0.04 and 0.78 ± 0.01, respectively. Therefore, the secondary structures in HMR3 and HMR2 simulations are consistent. Our previous SM simulations reported the overall helical fraction ⟨*H*⟩ = 0.63 ± 0.05, with Ct showing an almost 2-fold higher propensity (0.81 ± 0.05) than Nt (0.48 ± 0.08).^34^ Thus, even with incomplete equilibration, SM helical fractions, including those in Figure 3, agree with those of HMR3 and HMR2 when sampling errors are accounted.

**Figure 3:**
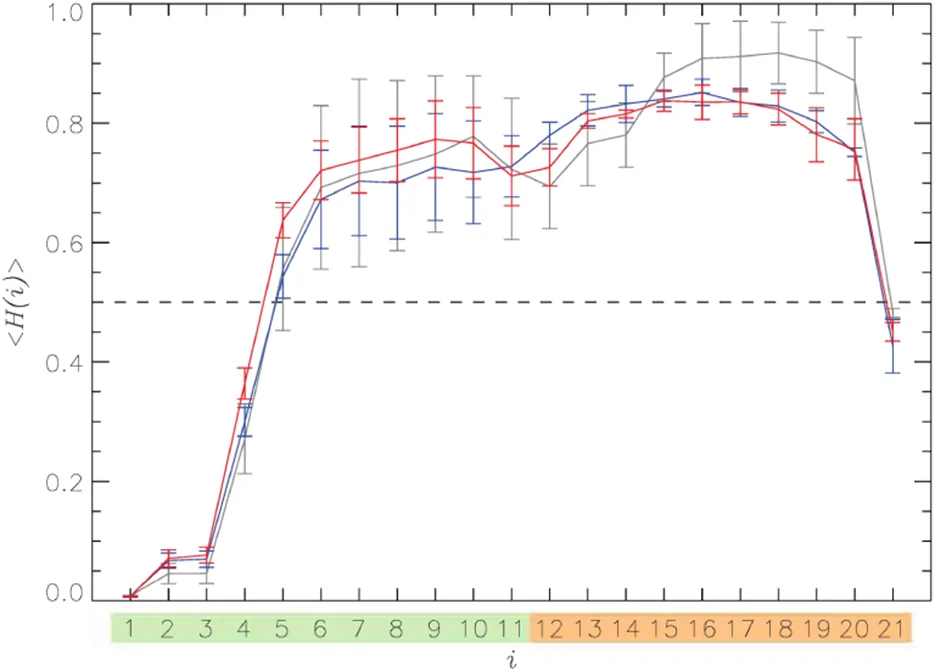
Distribution of helical propensities ⟨*H*(*i*)⟩ along PGLa amino acids *i*. Data in red, blue, and gray refer to HMR3, HMR2, and SM, respectively.^34^ Vertical bars indicate sampling errors. The N- and C-termini of PGLa are colored following Figure 1a.

In general, the formation of a stable helical structure in PGLa bound to a lipid bilayer is expected due to its strong hydrophobic moment μ.^33^ Because the distribution of PGLa amino acids is highly amphiphilic, its C-terminus has μ = 8.4 kcal/mol, which is reduced to 3.3 kcal/mol in the Nt. The computations of the hydrophobic moment explain the stronger helical propensity in the PGLa C-terminus than in the Nt. Importantly, simulation results are in excellent agreement with the NMR spectroscopy data. Indeed, the PGLa peptide interacting with DPC micelles acquires a helical state at amino acid positions *i* = 6–21.^25^ CD experiments also showed that when PGLa binds to POPC/POPG vesicles, its ⟨*H*⟩ ∼0.8.^44^ Similar results follow from Raman and Fourier spectroscopy analyses.^45,46^

According to Figure 2b the center of mass of the PGLa peptide in HMR3 (HMR2) simulations at *t* > *t*_eq_ is positioned at the distance ⟨*z*_CM_⟩ = 10.9 ± 0.4 Å (10.8 ± 0.4 Å) from the bilayer midplane. Therefore, the peptide is deeply inserted into the bilayer. To gather additional insight, we computed the probability distributions *P*(*z*,*i*) for amino acids *i* to occur at a distance *z* from the bilayer midplane. Figure 4a unequivocally shows that all amino acids in HMR3 simulations can be classified as inserted, because the average positions of their centers of mass ⟨*z*(*i*)⟩ occur below the phosphorus atoms, i.e., for all *i* ⟨*z*(*i*)⟩ < *z*_*P*_ (see Models and Methods). The same conclusion follows from HMR2 findings shown in Figure 4b. It is notable that in both panels, HMR and SM ⟨*z*(*i*)⟩ are in approximate agreement. Reflecting the adoption of a helical structure, Figure 4 demonstrates a clear periodicity in ⟨*z*(*i*)⟩ variations, in which the charged lysines *i* = Lys5, Lys12, Lys15, and Lys19 are located closer to the lipid phosphorus atoms to maintain electrostatic interactions. In contrast, the apolar amino acids *i* = Ala10, Ile13, and Ala17 are inserted far deeper. Thus, in both HMR simulations, the apolar side of the PGLa helix is facing the bilayer hydrophobic core, and its polar side is directed toward the lipid headgroups. If the acquisition of the helix is driven by the hydrophobic moment and the water–bilayer interface, then the helical propensity ⟨*H*⟩ may change with the depth of PGLa insertion into the bilayer. Figure S5 in Supporting Information confirms this expectation, showing that for HMR3 and HMR2, ⟨*H*⟩ reaches a maximum when the peptide’s center of mass is positioned *z*_CM_ ∼5 Å from the bilayer midplane. However, the figure also reveals that deeper insertion into the bilayer disrupts helical structure. A similar observation follows from the SM data. Furthermore, according to Table 1, the equilibration time scales probing insertion are almost twice as fast as those for helix formation. Together, these findings suggest that PGLa helix acquisition is driven by the hydrophobic moment created by charged amino acids and the water–bilayer interface. If so, the PGLa helix formation mechanism is different from that in Aβ peptides, which can adopt a helical structure in a transmembrane position.^47^ In contrast to PGLa, Aβ does not need a water–bilayer interface and can form a helix by locking backbone hydrogen bonds in the hydrophobic medium created by lipid tails.

**Figure 4:**
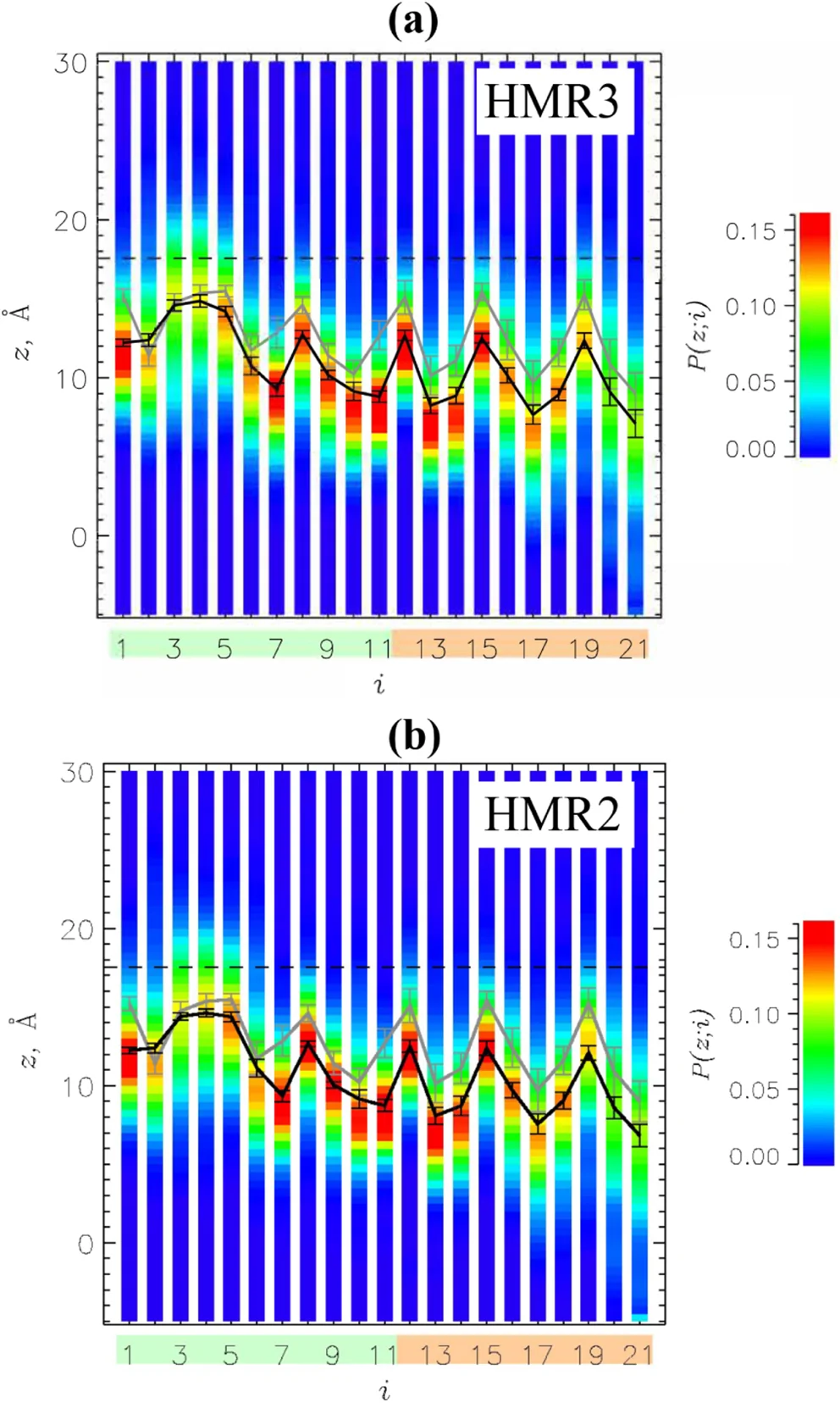
Probability distributions *P*(*z;i*) map the positions *z* of PGLa amino acids *i* along the bilayer normal in HMR3 (a) and HMR2 (b) simulations. *P*(*z;i*) values are color-coded according to the scale. In both panels, black and gray lines trace the average positions of amino acid centers of mass ⟨*z*(*i*)⟩ in HMR and SM^34^ models, respectively. The average position of the center of mass of phosphorus atoms in a leaflet, *z_P_* = 17.56 Å, is marked by a dashed line. PGLa N- and C-termini are distinguished by colors following Figure 1a.

Additional information about the position of PGLa follows from the computations of the tilt angle γ, which measures peptide’s inclination (see Models and Methods). Consistent with a flat position of PGLa inferred from Figure 4, the HMR3 distribution *P*(γ) in Figure 5a is unimodal, with the average ⟨γ⟩ = 101 ± 1°. The data for HMR2 in Figure 5a is almost indistinguishable from the HMR3 counterpart (⟨γ⟩ = 101 ± 2°). It is worth mentioning that in our previous SM simulations, the average ⟨γ⟩ = 94 ± 2°.^34^ The probability distributions *P*(β) of the rotation angle β, measuring the positioning of Lys12 in HMR models, are presented in Figure 5b. In HMR3 simulations, this distribution is unimodal, with the mean ⟨β⟩ = 110 ± 2°. HMR2 data yield a value of ⟨β⟩ = 111 ± 1°, which within the error range of HMR3. Similar to Lys12, the side chain of Lys19 is oriented upward, with the average rotation angles being 91 ± 3° in HMR3 and 100 ± 4° in HMR2. In SM, ⟨β⟩ for Lys12 and Lys19 were 96 ± 11 and 125 ± 21°.^34^ Thus, in both HMR simulations, the PGLa peptide is inserted in the bilayer below the phosphorus atoms, almost parallel to the bilayer surface, with lysine side chains pointed upward. According to Figure 5 mass repartitioning data are in approximate but not perfect agreement with the SM simulations (see below).

**Figure 5:**
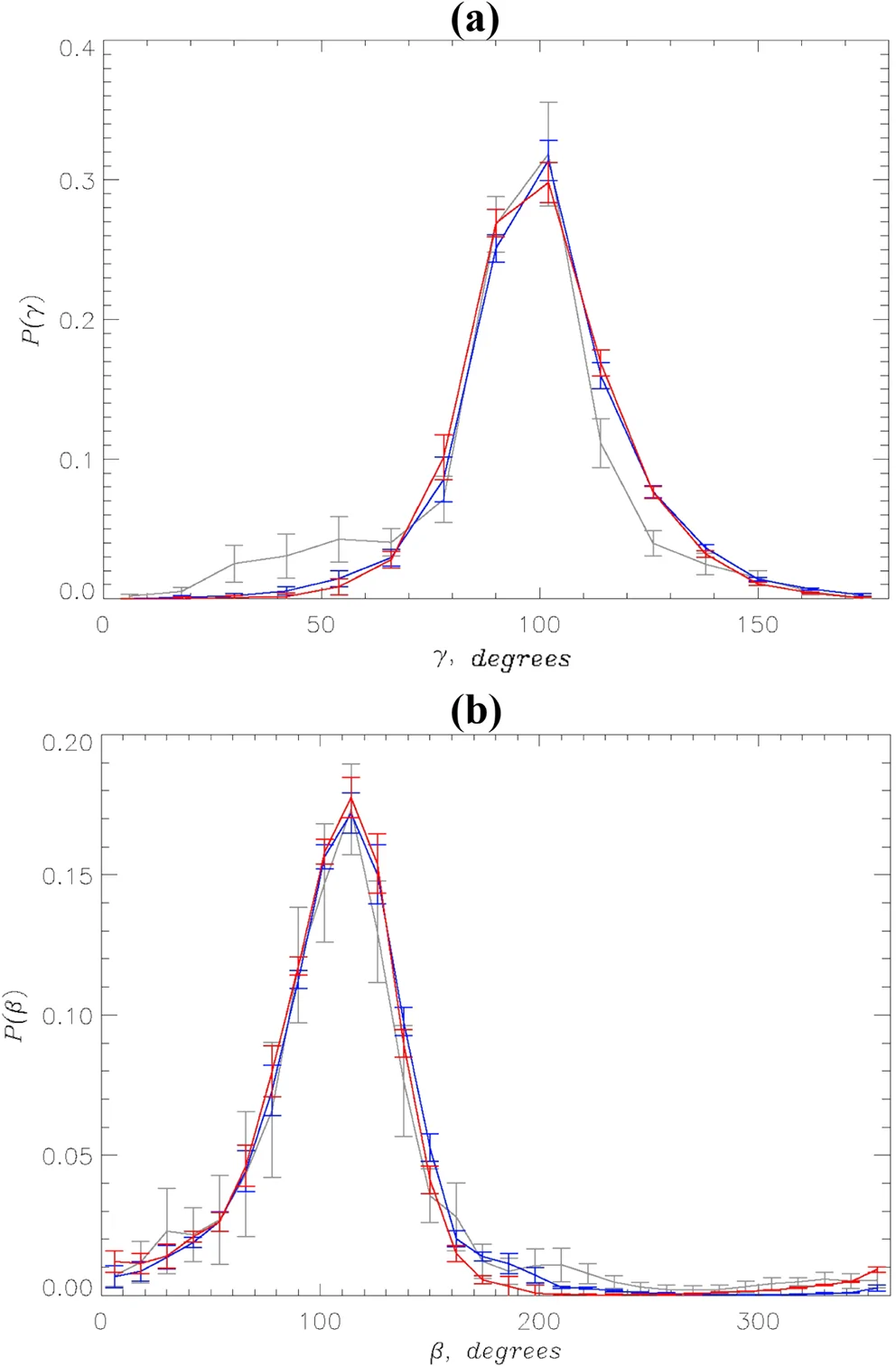
(a) Probability distributions *P*(γ) of the PGLa tilt angles γ. (b) Probability distributions *P*(β) of Lys12 rotation angles β. In both panels, HMR3, HMR2, and SM^34^ data are in red, blue, and gray, respectively.

The analysis of Lys rotation angles and positioning of PGLa deduced from Figure 4 suggests that Lys charged side chains maintain electrostatic interactions with phosphate lipid groups. To test this expectation for HMR simulations, we computed the numbers of contacts ⟨*C*_l_(*i*)⟩ and ⟨*C*_P_(*i*)⟩ formed by amino acid *i* with all lipid groups and with phosphate (P) groups, respectively. The resulting contact vectors are plotted in Figure 6a, which shows PGLa positively charged amino acids Gly1, Lys5, Lys12, Lys15, and Lys19 forming most of the interactions with P groups. For example, in HMR3, these amino acids account for 6.1 contacts out of 10.0 formed by all amino acids with P. Furthermore, the total number of PGLa-lipid contacts ⟨*C*_l_⟩ is 45.8, of which 14.5 are contributed by the charged amino acids. Thus, these residues deliver almost two-thirds (61%) of all interactions with phosphate groups and 32% of all peptide-lipid interactions while constituting 24% of the PGLa sequence. HMR3, HMR2, and SM^34^ contact vectors are in good agreement, being well within the sampling errors. Thus, charged PGLa amino acids serve as anchors binding the peptide to the bilayer.

**Figure 6:**
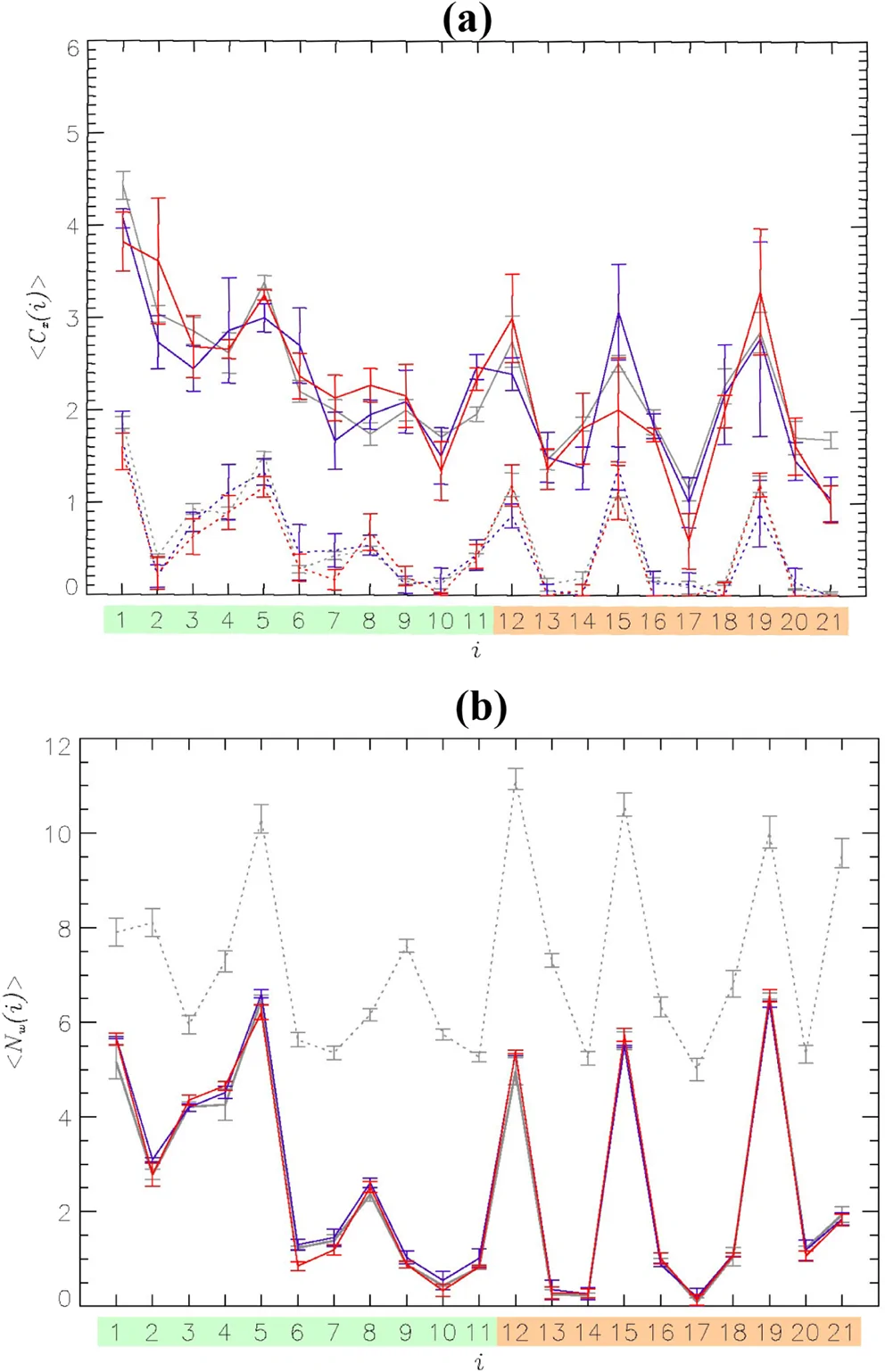
(a) The contact vector ⟨*C*_l_(*i*)⟩ reports the number of contacts between amino acid *i* and lipid groups, whereas ⟨*C*_P_(*i*)⟩ restricts these interactions to anionic phosphate lipid groups. Solid and dashed lines represent ⟨*C*_l_(*i*)⟩ and ⟨*C*_P_(*i*)⟩, respectively. (b) The hydration vector ⟨*N*_w_(*i*)⟩ reports the number of water molecules in the first solvation shell of amino acid *i*. The black dotted line in (b) represents ⟨*N*_w_(*i*)⟩ computed for PGLa in lipid-free water (see SI). In both panels, data in red, blue, and gray correspond to HMR3, HMR2, and SM simulations. PGLa regions are distinguished by colors following Figure 1a. Sampling errors are given by vertical bars.

Since the peptide is positioned in the bilayer core well below the lipid headgroups, we examined its dehydration as described in Models and Methods. It follows from the simulations of PGLa in water (see SI) that the first solvation shell of the peptide contains ⟨*N*_w_⟩ = 114 water molecules. Upon insertion into the bilayer, ⟨*N*_w_⟩ is reduced to 39 ± 0 in HMR3 or 40 ± 1 in HMR2, both implicating the loss of two-thirds of hydrating water. Due to the orientation of PGLa in the bound state, hydration may be uneven along the sequence. This conclusion is borne out in Figure 6b, which displays dramatic variations in the hydration vector, a number of waters ⟨*N*_w_(*i*)⟩ present in the first solvation shells of amino acids *i*. All Lys and Gly1 are solvated by 5 to 6 water molecules, whereas hydrophobic *i* = Ile9, Ala10, Gly11, Ilu13, Ala14, and Ala17 are almost completely dehydrated, as their ⟨*N*_w_(*i*)⟩ < 1. The latter amino acids are positioned on the apolar helix side deep in the bilayer core. In contrast, positively charged amino acids are proximal to lipid headgroups and the water–bilayer interface. However, as already implied by ⟨*N*_w_⟩, the bound PGLa experiences significant dehydration, as even these charged amino acids lose 41% of water molecules from their solvation shells. Figure 6b indicates that HMR3, HMR2, and SM hydration vectors are almost indistinguishable.

### 3.3 Disorder in Bilayer Structure Caused by PGLa Binding

The disorder in the DMPC/DMPG bilayer caused by PGLa binding is mapped by the volume number density *n*_v,l_(*r*,*z*) of the lipid heavy atoms as a function of the distance *r* to the PGLa center of mass and the distance *z* to the bilayer midplane (see Models and Methods). Figure 7 compares the two *n*_v,l_(*r*,*z*) values computed using the HMR3 and HMR2 models. Visual inspection reveals their good agreement, with both exhibiting a profound dip in the lipid density displaced by the bound peptide. Following Models and Methods, we defined the bilayer thinning Δ*D* = *D*_dist_ – *D*_near_, where *D*_dist_ and *D*_near_ are the bilayer thicknesses in the distant and near regions. According to Figure 7 Δ*D* is 19.3 ± 0.3 Å for HMR3 and 20.0 ± 0.0 Å for HMR2, i.e., these values are very close. The bilayer thinning obtained in HMR3 and HMR2 is more pronounced than Δ*D* = 17.3 ± 0.3 Å reported for SM;^34^ the reason for this difference is clarified in the next section.

**Figure 7:**
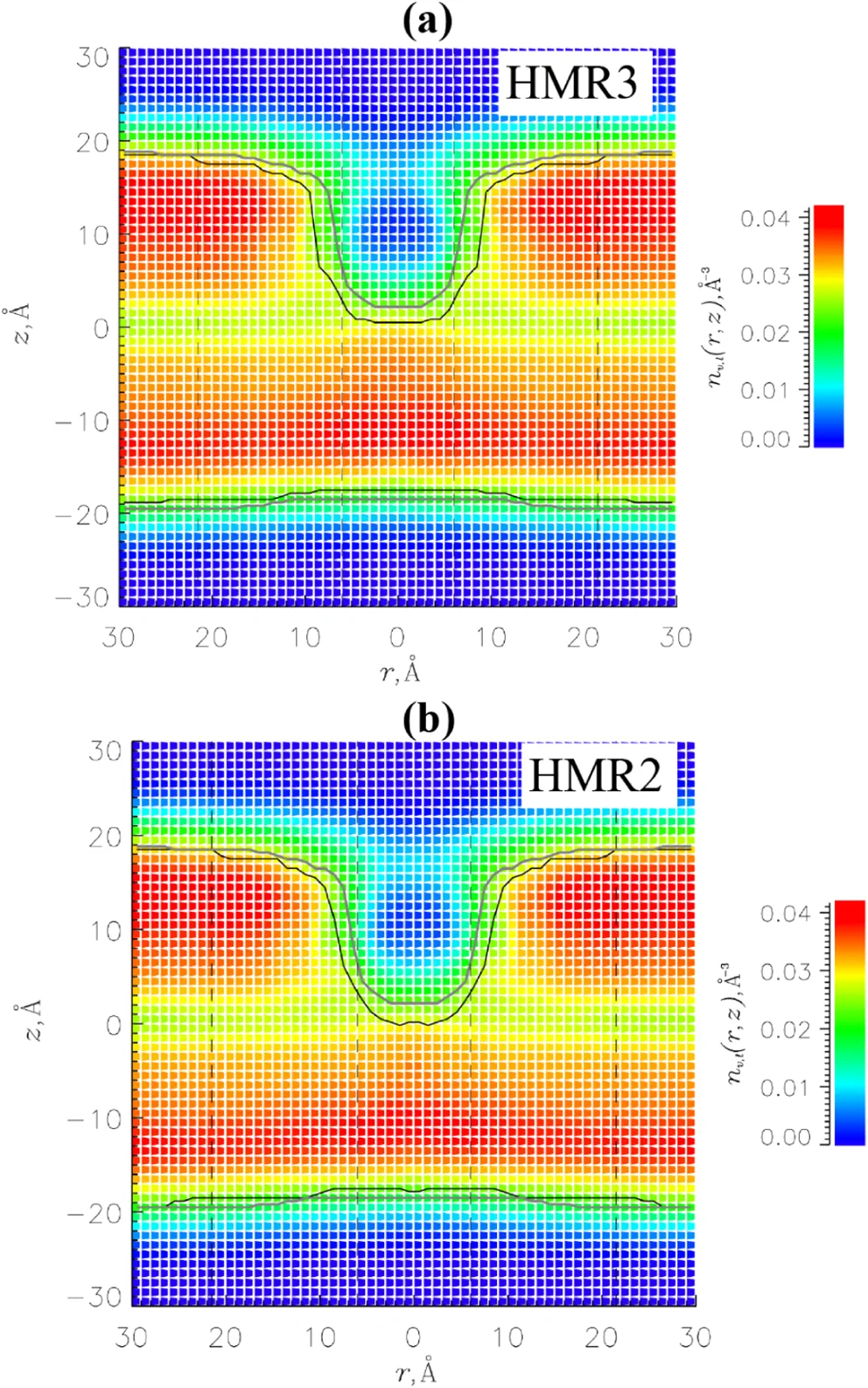
Volume number density of lipid heavy atoms *n*_v,l_(*r,z*) plotted as a function of the distance *r* to the PGLa center of mass and the distance *z* to the bilayer midplane. Panels (a) and (b) compare *n*_v,l_(*r,z*) for the HMR3 and HMR2 models, respectively. In both panels, continuous black and gray lines represent the bilayer boundaries *z*_b_(*r*) in HMR and SM models, respectively; the dashed lines distinguish distant and near bilayer regions.

In our previous studies, we examined the depletion of lipid density around the bound peptide.^34^ To ascertain that mass repartitioning reproduces these findings, we computed the lipid surface number densities, *n*_s,x_ (see Models and Methods). The results are presented in Table 2. It is seen that for HMR3, *n*_s,l_, which measures the density of all lipids, decreases by a factor of 2.4 between the distant and near regions. A minor decrease in the DMPG surface density between these regions is evident, whereas there is a dramatic 3.7-fold drop in the corresponding DMPC density, *n*_s,DMPC_. Although, due to the finite size effects, the DMPC:DMPG ratio in the distant region slightly deviates from the expected 3:2, it becomes inverted to 3:4 in the near region. Nearly the same results follow from the HMR2 model. According to Table 2, the findings for both HMR models are consistent with our previous SM data.^34^ Thus, HMR3 and HMR2 models demonstrate (i) bilayer thinning, (ii) depletion of lipids in the peptide binding footprint, and (iii) relative influx of DMPG and efflux of DMPC lipids from the near region. Similar results have been observed in our previous studies, which did not utilize mass repartitioning.^34^ Congregation of DMPG lipids near bound PGLa peptides resembles the PGLa-induced clustering of anionic lipids reported in differential scanning calorimetry experiments.^48^ Earlier X-ray reflectivity measurements of lipid monolayers also pointed to the mixing of PGLa with anionic lipids while partitioning from zwitterionic ones.^49^

**Table 2:** Lipid Surface Density Distributions

	*n*_s,l_,	*n*_s,DMPG_,	*n*_s,DMPC_,
bilayer region	10^–2^ Å^–2^	10^–2^ Å^–2^	10^–2^ Å^–2^
HMR3			
Near	0.68 ± 0.01	0.40 ± 0.02	0.28 ± 0.05
Distant	1.60 ± 0.00	0.58 ± 0.01	1.03 ± 0.01
HMR2			
Near	0.69 ± 0.04	0.41 ± 0.01	0.29 ± 0.04
Distant	1.61 ± 0.00	0.59 ± 0.01	1.02 ± 0.01
SM^34^			
Near	0.84 ± 0.07	0.56 ± 0.03	0.28 ± 0.05
Distant	1.62 ± 0.01	0.58 ± 0.01	1.05 ± 0.01

### 3.4 Binding Free Energy Landscape

Our previous analysis of PGLa binding based on SM model^34^ has used the two-dimensional free energy landscape *G*(*z*_CM,Ct_,β) computed as a function of the insertion of the peptide C-terminus *z*_CM,Ct_ and the Lys19 rotation angle β. That analysis identified two peptide-bound states, a dominant inserted state **I** and a metastable surface-bound state **SB**. In **I**, the peptide C-terminus is inserted deep into the bilayer hydrophobic core, positioned at ∼9.5Å from the midplane, with Lys19 directed upward and showing the rotation angle β ∼78°. In **SB**, PGLa is located at *z*_CM,Ct_ ∼25.5 Å with β ∼306°. Thus, in **SB**, PGLa is bound to the bilayer surface, and Lys19 points approximately down toward the bilayer midplane. The probabilities of **I** and **SB** were 0.82 and 0.07, respectively.

To check if HMR3 and HMR2 reproduce that free energy landscape, we plotted the respective *G*(*z*_*CM,Ct*_,β) in Figure 8. Comparison of mass-repartitioned and SM free energy landscapes reveals one unambiguous distinction—a complete or nearly complete absence of **SB** in HMR3 or HMR2. Both of these models feature a sole free energy basin **I** at *z*_CM,Ct_ ∼10 Å and β ∼90° that approximately matches the inserted state **I** from SM. The appearance of this sole state in HMR models is consistent with the deep PGLa insertion observed in Figure 2b and the computation of Lys19 rotation angles above. In our previous paper,^34^ we determined the probability of state **I** by including all microstates in the range [*G*_min_,*G*_min_ + Δ*G*], where *G*_min_ is the state free energy minimum and Δ*G* = 2.8 kcal/mol is the maximum increase in *G* along the minimum free energy path from **I** to **SB**. If the same definition is applied to HMR3, state **I** in Figure 8a occurs with the probability of *P*(*I*) = 0.95 ± 0.02 and features a high helical content *H* = 0.72 ± 0.02. The peptide center of mass is positioned at *z*_CM_ = 9.5 ± 0.0 Å. Very similar results follow from the analysis of HMR2 **I** in Figure 8b (*P*(*I*) = 0.92 ± 0.02, *H* = 0.71 ± 0.01, and *z*_CM_ = 9.5 ± 0.1 Å).

**Figure 8:**
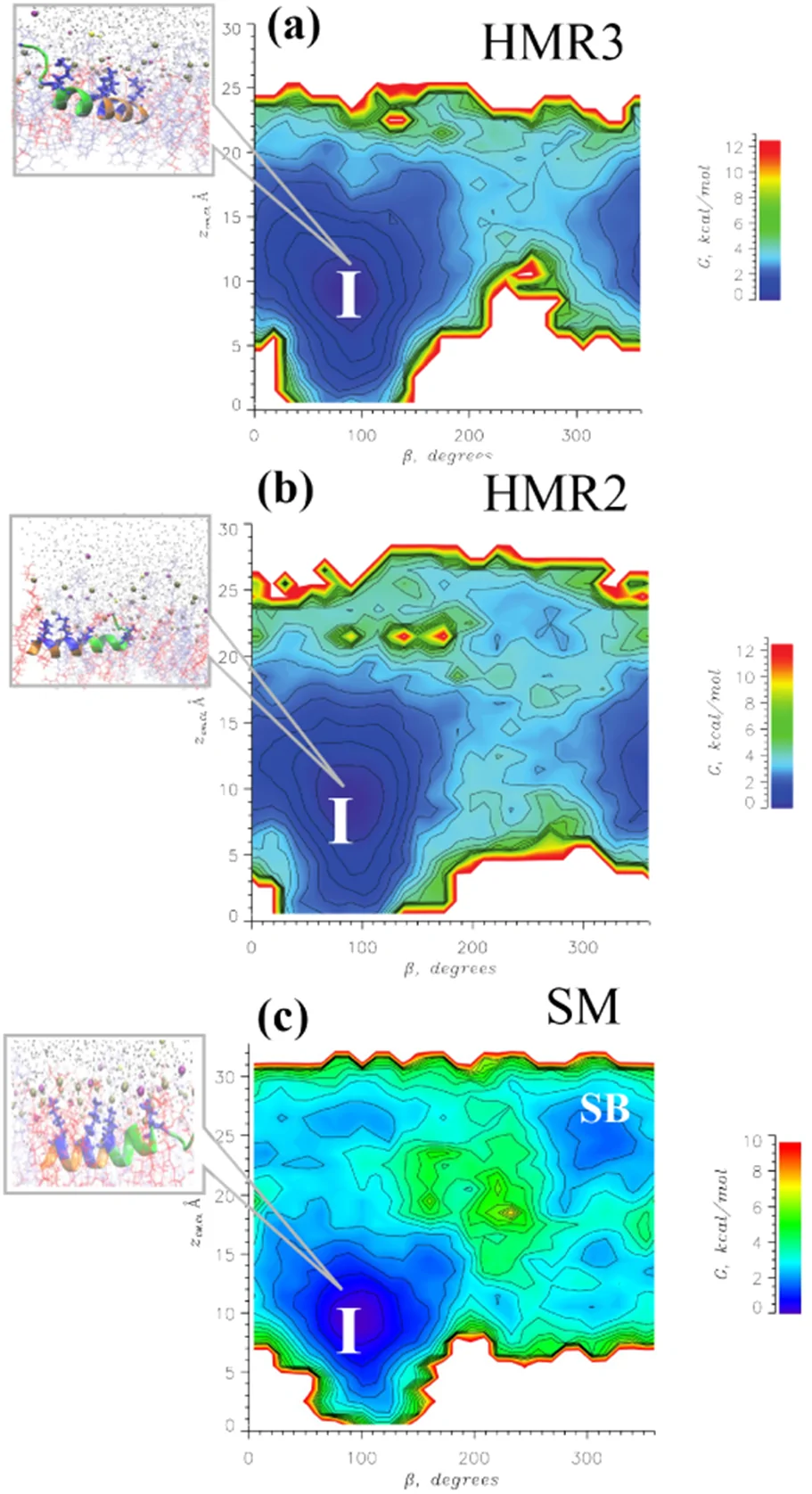
Free energy landscape *G*(*z_CM,Ct_*,β) governing PGLa binding to the DMPC/DMPG bilayer is presented in terms of the C-terminus center of mass insertion *z*_CM,Ct_ and the Lys19 rotation angle β. The panels (a)–(c) are computed for HMR3, HMR2, and SM models. The contour lines have increments of 0.5 kcal/mol. Representative PGLa structures from the inserted **I** state are shown for HMR models. The depiction of molecules follows Figure 1c. The SM landscape has been computed previously^34^ and is included for reference.

An apparent disappearance of the surface-bound state **SB** in Figure 8 is a consequence of better peptide equilibration in the bilayer in the mass repartitioning models. To substantiate this assertion, we tracked the appearance of **SB** along the REHT simulation timeline (see SI). We partitioned the SM and HMR simulations into four or six 100 ns batches, each collecting 300 ns of sampling across three REHT trajectories. For each batch, we computed the probability distribution *P*(*z*_CM,Ct_) of the position of the PGLa C-terminus center of mass *z*_CM,Ct_ along the bilayer normal (Figure S6). To define **SB** on the basis of *P*(*z*_CM,Ct_), we selected a cutoff insertion depth *z*_c_ = 16 Å that reproduces the probability of the **I** state in HMR3 simulations at equilibrium, i.e., *P*(*I*) = 0.95. Assuming that **SB** occurs at *z*_CM,Ct_ > *z*_c_, we applied this definition to all batches and simulations and computed the probabilities of the **SB** state *P*(SB) in Table S2. This table and Figure S6 unequivocally show that **SB** appears early in all simulations, SM or HMR, but then gradually diminishes with equilibration. Due to longer sampling times, HMR simulations register a nearly complete disappearance of **SB** by the end of REHT trajectories, while it still lingers in SM. Based on this evidence, we believe that **SB** is an intermediate along the PGLa insertion path into the bilayer. Similar evolution of this intermediate in SM and HMR simulations also supports the sampling consistency across the models.

Because **SB** in SM is relatively rare, being about 10-fold less probable than **I**, HMR simulations generally reproduce the findings collected with the standard masses. The benefit of the HMR simulations lies in refining the conformational ensemble of the bound PGLa by excluding a metastable surface-bound state. This correction improves the consistency between our REHT findings and the experiments. Indeed, the tilt γ and rotation β angles observed in HMR3 compare better with the experimental values than SM findings. In the experiments, a PGLa peptide was bound to a DMPC/DMPG bilayer at 308 K, full hydration, and a low 1:200 peptide-to-lipid ratio.^27,31^ Under those conditions, the experimental tilt angle is 98°, and HMR3 ⟨γ⟩ = 101 ± 1° is almost equal to the experimental result. In SM, ⟨γ⟩ = 94 ± 2°,^34^ which is farther away from the experimental value. Furthermore, the HMR3 rotation angle ⟨β⟩ for Lys12 is 110 ± 2°, and it is notably closer to the experimental 115° than ⟨β⟩ = 96° computed in SM.^34^ Similar observations apply to HMR2. Finally, the experiments at low peptide-to-lipid ratio have resolved only one state, S, in which PGLa lysine side chains are pointed upward.^27,28^ This state is inconsistent with **SB** from the SM simulations but is in excellent agreement with **I** in HMR.

### 3.5 Are Mass-Repartitioned Models Computationally Efficient?

The previous sections showed that mass repartitioning affords better equilibration in the complex biomolecular system. The sampled conformational ensembles produced by HMR3 and HMR2 models are in excellent agreement, and the structural quantities collected with and without mass repartitioning are also consistent if corrected for system equilibration. However, the principal question remains unanswered—are HMR models computationally more efficient than the SM model? To address this question, we consider the equilibration time scales τ extracted from the exponential fits in Table 1. The time scales τ measuring penetration of PGLa into the bilayer differ by no more than 12%. The time scales τ reflecting helix formation in SM and HMR exhibit larger variations of up to 28%.

To measure computational efficiency, i.e., the amount of computation, we introduced the time scaled by the integration step-up factor σ (see Models and Methods). The resulting time scales τ_σ_ = τ/σ are listed in Table 1. Their comparison shows that both HMR3 and HMR2 models are 3.6-fold more efficient than SM in equilibrating peptide insertion. However, with respect to helix equilibration, HMR3 and HMR2 gains in efficiency are weaker, as they reduce the computational load by a factor of 3.5 or 2.8, respectively. Thus, the HMR3 computational advantage may be 13% lower than a straightforward theoretical gain by the factor of σ = 4. HMR2 shows the same trend of decreased gains but with less consistency. Although the HMR3 conclusions should be viewed with caution due to sampling errors in time scales τ, they are consistent with the previous reduction estimate of 15%.^19^ The variations in equilibration time scales are likely to be linked to hydrogen-bond-rich states, such as helix, which depend on hydrogen kinetics. In support of this conclusion, our recent simulations testing equilibration of alanine dipeptide with the HMR3 model did not reveal consistent variations in time scales τ_σ_ measuring transitions between dihedral angle states.^22^ We note that, opposite to PGLa, alanine dipeptide has no secondary structure. Thus, complex biomolecular systems may reduce the efficiency of hydrogen mass repartitioning.

The comparison of computational efficiencies is affected by many factors beyond the step-up factor or hydrogen kinetics. Among those are the frequency of computing long-range (LR) interactions. Following previous studies,^19^ we did not adjust LR computations with the integration step δ*t*. Therefore, the neighbor lists were updated every *f* = 20 integration steps, with long-range (LR) electrostatic interactions evaluated every second step. However, some previous investigations adjusted *f* depending on δ*t*, e.g., by setting it to 5 for HMR3.^21^ Therefore, to assess this effect on computational efficiency, we performed test REHT simulations with *f* = 10 and also computed LR electrostatics every integration step. We found that these changes slowed down HMR3 and HMR2 simulations by about a factor of 1.2. The corrected time scales *τ*_*σ*,cor_ shown in Table 1 indicate that the HMR3 gain factor in computational efficiency is thereby reduced to about 2.9.

However, the necessity to adjust the frequency of neighbor list updates depends on the default settings in SM. For example, NAMD applies the default *f* = 20 if δ*t* = 1 fs. We have examined NAMD logs for the warning messages reporting recalculation of neighbor lists due to large displacement of atoms. We found that in HMR3 simulations with δ*t* = 4 fs and *f* = 20, the neighbor lists are recomputed only 0.8% more frequently than at δ*t* = 1 fs. This finding suggests that the choice of *f* = 20 is highly conservative, and HMR3 simulations may be executed without adjustments in *f* as done in our study. Therefore, the gains in HMR3 computational efficiency applicable to NAMD may be estimated without correcting for the frequency of neighbor list computation.

Our analysis indicates that the HMR3 actual gain in computational efficiency is ∼3.5. Clearly, the performance of SM can be readily improved by increasing the integration step size to 2 fs. This option does not affect atom masses and can be used with NAMD without adjusting the computation of LR interactions, thereby increasing computational efficiency 2-fold. However, it still does not match the performance of HMR3, suggesting that the latter is still superior in boosting conformational sampling.

## 4 Conclusions

In this paper, we tested two mass repartitioning models, HMR3 and HMR2, with tripled and doubled hydrogen masses, against the model with standard masses (SM). HMR3 and HMR2 utilized longer integration steps of 4 and 3.5 fs, respectively, whereas SM used a 1 fs time step as a reference. As a case study, we took the REHT simulations of antimicrobial peptide PGLa binding to an anionic DMPC/DMPG bilayer. Comparing two different mass repartitioned models allowed us to check for any potential sampling biases. With respect to our previous SM simulations, we extended sampling by 50%, collecting across all conditions 36 μs for each model. Our investigations led to three main conclusions. First, longer simulations afforded by HMR3 and HMR2 models better equilibrate PGLa peptide binding to the lipid bilayer. Specifically, they eliminated a metastable surface-bound state **SB** observed in SM. In mass repartitioned models, PGLa exclusively samples the inserted state **I**, which also appeared as a dominant state in SM but alongside **SB**. This better equilibration of peptide binding improved the agreement with experimental data. Second, HMR3 and HMR2 reproduced a broad range of structural properties previously reported for SM after correcting them for equilibration. In addition, both mass repartitioning models demonstrate excellent agreement between themselves. These findings suggest that mass repartitioning models preserve structural properties in this complex biomolecular system. Third, the actual acceleration of sampling offered by HMR3 is reduced from the theoretical 4-fold to about 3.5-fold. This effect may be due to the slow dynamics of heavy hydrogens. HMR2 shows qualitatively similar results. An increase in the integration step length in SM to 2 fs or adjustments in the computations of long-range interactions reduce the computational gains offered by mass repartitioning but do not change the overall outcome. HMR2 and particularly HMR3 are excellent options to boost conformational sampling in complex biomolecular systems.

## Supplementary Material



## Data Availability

NAMD is available at https://www.ks.uiuc.edu/Research/namd/. VMD is available at https://www.ks.uiuc.edu/Research/vmd/. Initial structures, topology files, NAMD configuration files, and codes used for data analysis are available at https://github.com/srbowers5/PGLA_MEM.

## Notes

Additional details concerning the model, methods, and results are provided in Supporting Information. This information is available free of charge at the Web site (PDF)
