# Supplementary material for: Replica Exchange Molecular Dynamics with Hydrogen Mass Repartitioning Improves Sampling of Peptide Binding to a Lipid Bilayer
Source: J Chem Theory Comput. 2026 Aug 4;22(16):8657–68. doi: 10.1021/acs.jctc.6c01064 (PMC13523638; doi:10.1021/acs.jctc.6c01064)
Supplement: Supplementary file 1 [file ct6c01064_si_001.pdf]

Supporting Information

Replica exchange molecular dynamics with hydrogen mass repartitioning improves sampling of peptide binding to a lipid bilayer

Steven R. Bowers, Christopher Lockhart, and Dmitri K. Klimov
School of Systems Biology, George Mason University, Manassas, VA 20110
E-mail: dklimov@gmu.edu

Selecting peptide force field: Following our previous studies [1,2] we selected the all-atom CHARMM22 force field with CMAP corrections for PGLa peptides. Briefly, the rationale is based on our finding that consistent with the experiments [3] this force field rather than a more recent CHARMM36m [4] reproduces the PGLa helical state upon binding to the DMPC/DMPG bilayer.

REHT setup: We used replica exchange with hybrid tempering (REHT) molecular dynamics. The simulations utilized R=20 conditions (T_m,T'_m), where T_m and T'_m are the solute and effective solvent temperatures, and m is the condition index. The list of REHT conditions is given in Table S1.

Table S1: List of REHT conditions used in the simulations probing PGLa binding to DMPC/DMPG bilayer.

m	T_m	T'_m
**0**	310	310
**1**	315	312
**2**	321	314
**3**	326	316
**4**	332	318
**5**	338	320
**6**	344	322
**7**	350	324
**8**	356	326
**9**	362	328
**10**	368	330
**11**	375	333
**12**	381	335
**13**	388	337
**14**	395	339
**15**	401	341
**16**	408	343
**17**	415	346
**18**	423	348
**19**	430	350

REHT performance: REHT technical performance was checked following the approach from our previous studies [1,2]. REHT formalism prescribes random walks of replicas across the conditions. Fig. S1 illustrates these walks suggesting that replicas diffuse randomly across the conditions. This figure does not show obvious trapping of replicas at any condition.

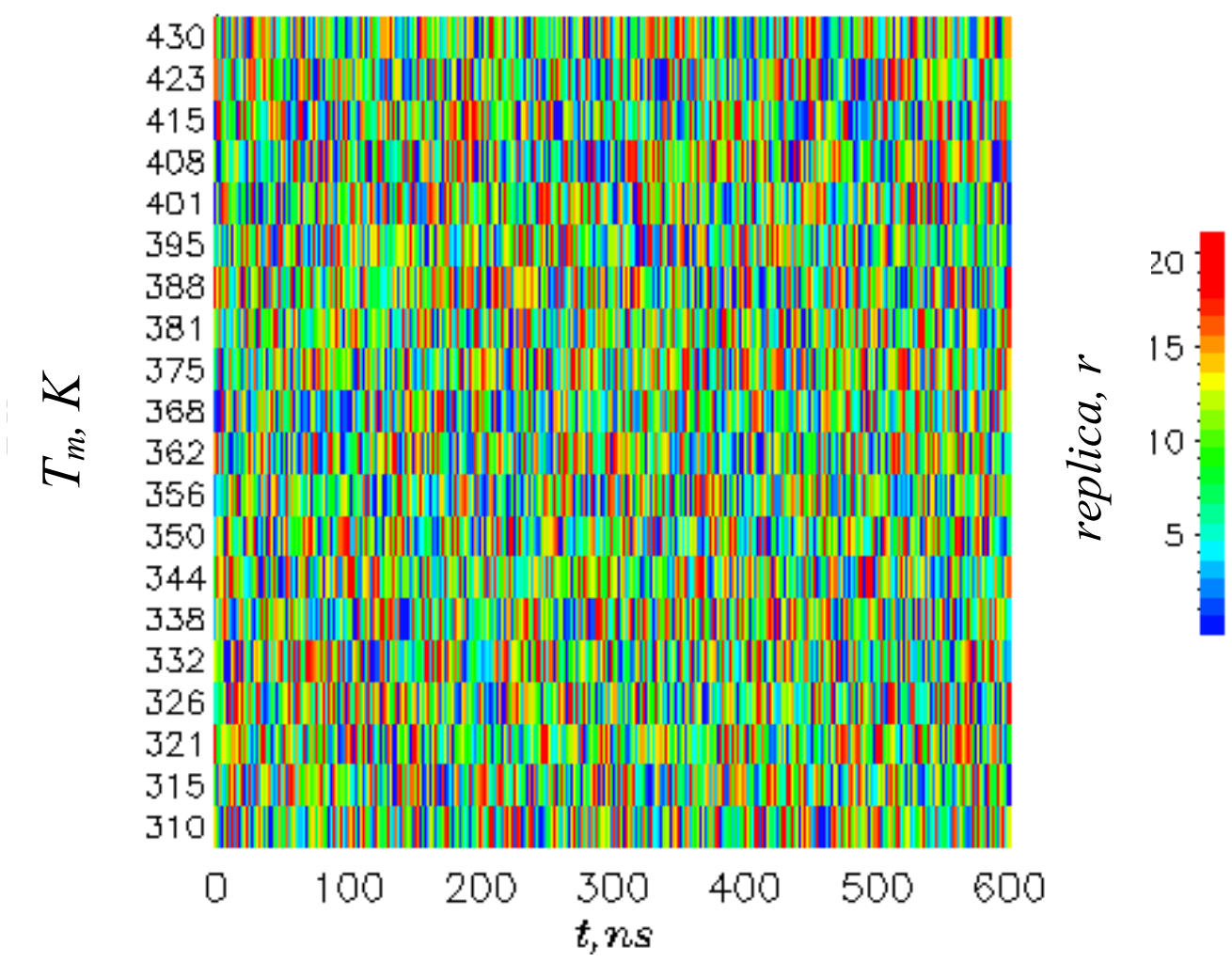

Figure S1 Replica walks across the conditions m (T_m,T'_m) in the representative REHT trajectory for HMR3 model. Each replica is assigned a unique color at the start of the trajectory according to the scale. Similar plots are seen for other HMR3 and HMR2 trajectories.

To provide a quantitative probe of replica distribution across the conditions, we used the replica mixing parameter [5,6],

$$h(m) = 1 - \frac{\sqrt{\sum_{r=0}^{R-1} t_r^2}}{\sum_{r=0}^{R-1} t_r}, \quad \text{(S1)}$$

where m is the REHT condition and t_r is the time spent by replica r at m. If REHT generates a random distribution of replicas over all R conditions, $h(m)$ reaches the constant maximum value $h_o = 1 - 1/R^{1/2}$. Since R=20, h_o is 0.78. For HMR3 Fig. S2 shows that $h(m)$ approaches h_o at all m with the average h across all conditions being 0.78. HMR2 replica mixing parameter behaves in a similar way. Finally, Fig. S3 presents the replica exchange rates $\alpha(m)$. For HMR3 and HMR2 models the average $\alpha(m)$ is 0.50 and 0.50, respectively. These rates are approximately optimal [7]. It follows from Figs. S1-S3 that REHT simulations produce a good mixing of replicas across the conditions as expected.

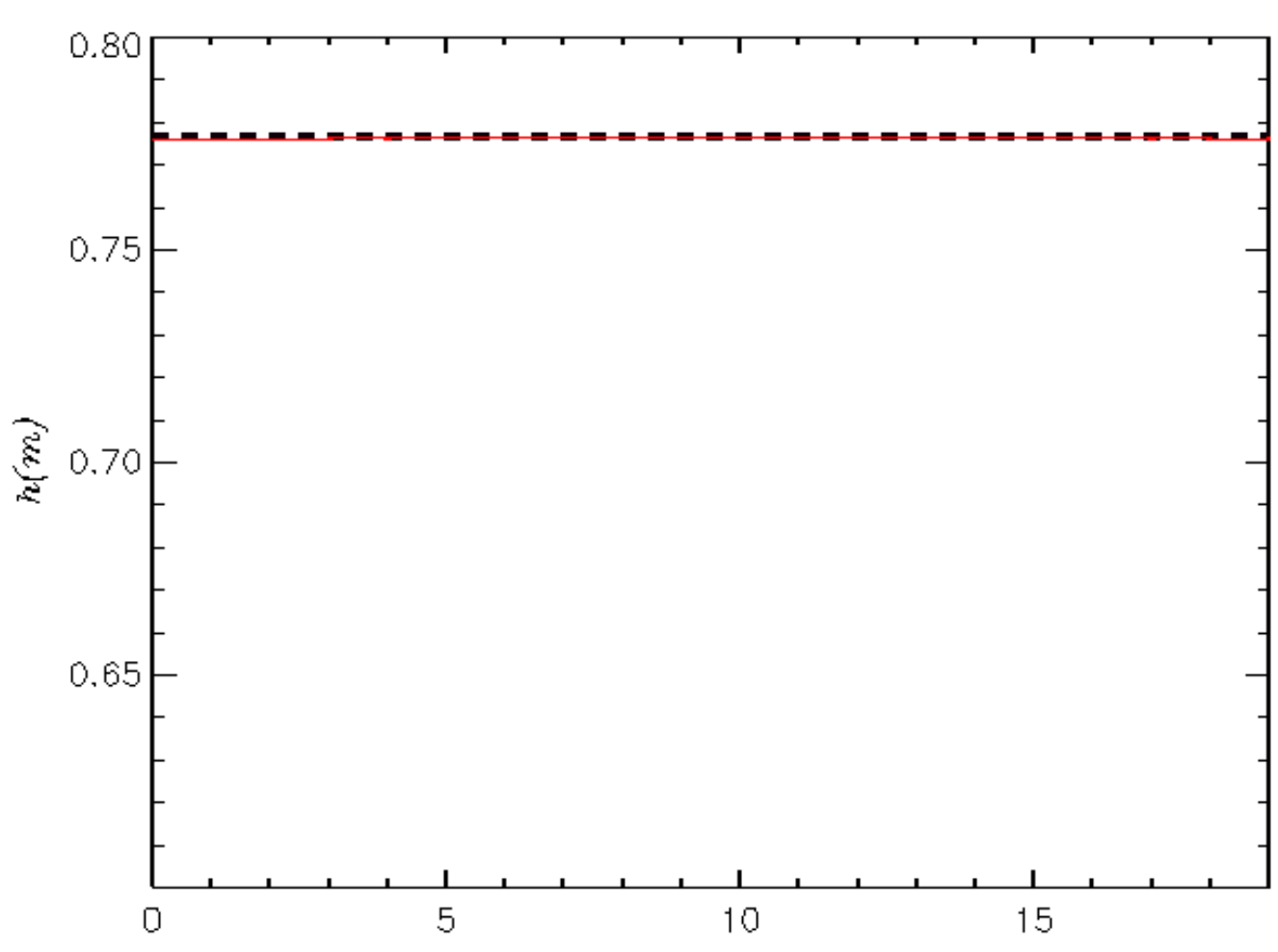

Figure S2 The replica mixing parameter $h(m)$ as a function of REHT condition m. Dashed thick line marks the maximum value h_o. HMR3 data is in red. HMR2 data is indistinguishable from HMR3. The data is averaged over REHT trajectories.

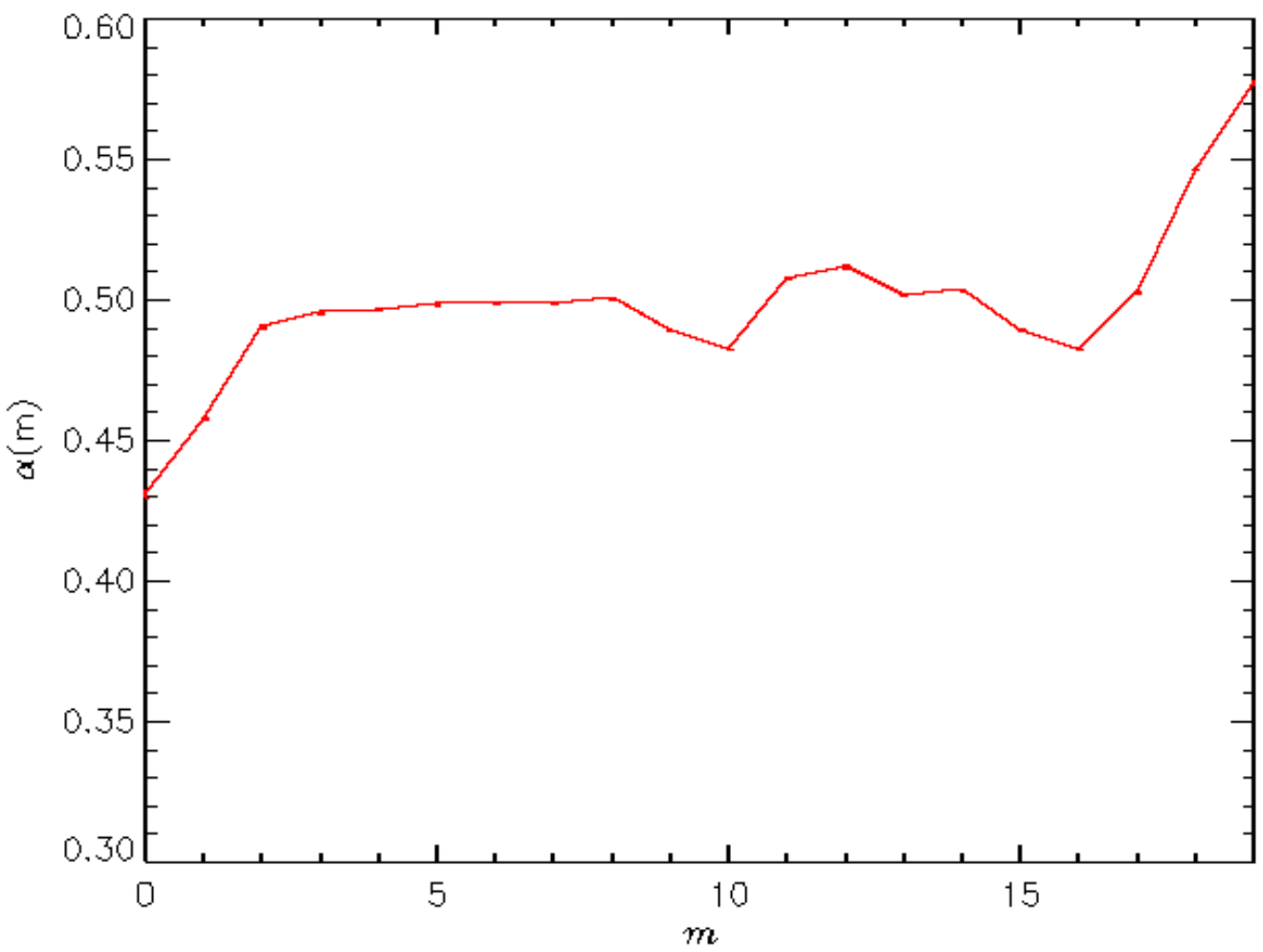

Figure S3 Replica exchange rates $\alpha(m)$ as a function of REHT conditions m. HMR3 data is in red. HMR2 data is indistinguishable from HMR3. The rates $\alpha(m)$ are averaged across REHT trajectories.

Initialization of HMR simulations: Fig. 2 shows a consistently lower helix propensity in HMR simulations compared to SM even though they shared the same initial conditions. An apparent reason for this discrepency is the initialization of HMR simulations with SM velocities. Due to lighter hydrogens the magnitude of SM velocities is larger than expected in HMR resulting in a helix structure disruption. In fact, without mass adjustment the instanteneous initial temperature at 310 K condition is 421 K in HMR2 and 555 K in HMR3 simulations. To further support this explanation, we performed short HMR3 simulations using HMR3 initial velocities, i.e., generated with the repartitioned masses. The results displayed in Fig. S4 show that the helical fraction *H(t)* in the HMR3 simulations initialized with the "proper" velocities follow SM *H(t)*. Therefore, the practical recommendation that follows from our analysis is to avoid initialization of HMR simulations with SM velocities. Ignoring this recommendation may cause partial helical and concievably other structural disordering that unnecessarily delays system equilibration.

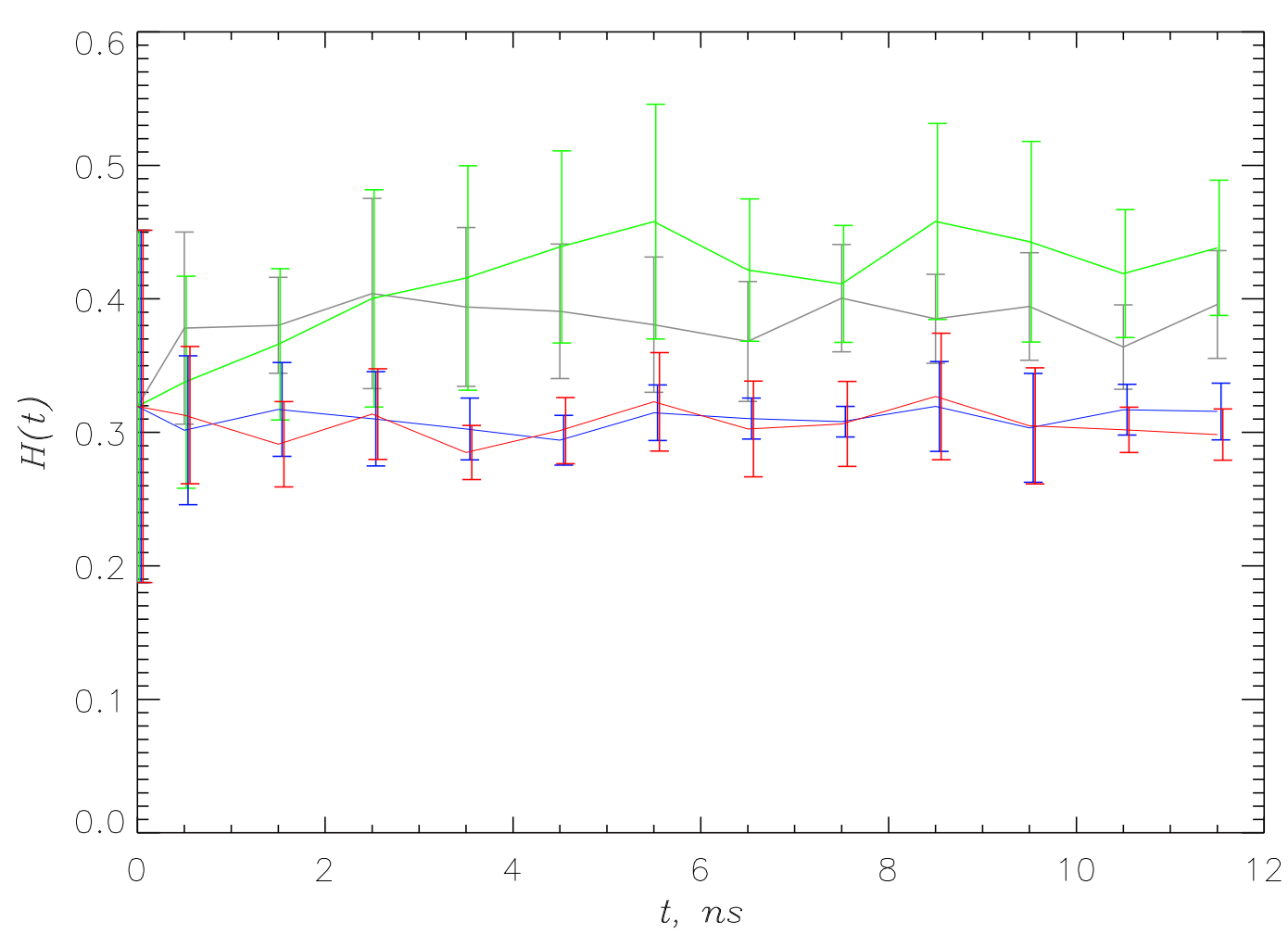

Figure S4 The PGLa helical fraction *H(t)* is plotted as a function of REHT sampling time *t*. This figure compares *H(t)* observed in SM (in grey), HMR3 (in red or green), and HMR2 (in blue) simulations at $t < 12$ ns. HMR3 simulations are initialized either with SM velocities (in red) or with the velocities reflecting mass repartitioning (in green).

Helical propensity changes with PGLa insertion: Since PGLa sequence harbors signficant hydrophobic moment, which causes helix formation upon peptide binding to the lipid bilayer, one may expect that its helical fraction <*H*> depends on the depth of penetration into the bilayer. Fig. S5 displays <*H*> as a function of the PGLa center of mass position z_{CM} along the bilayer normal. The figure demonstrates that helix fraction becomes maximum when the peptide deeply inserts into the bilayer, i.e., it resides in the **I** state. We show in the main text that in **I** PGLa charged side chains interact with phosphate groups, whereas apolar helix side faces lipid hydrophobic core. This peptide position accomodates amphiphilic helix the best explaining the drop in <*H*> near bilayer midplane.

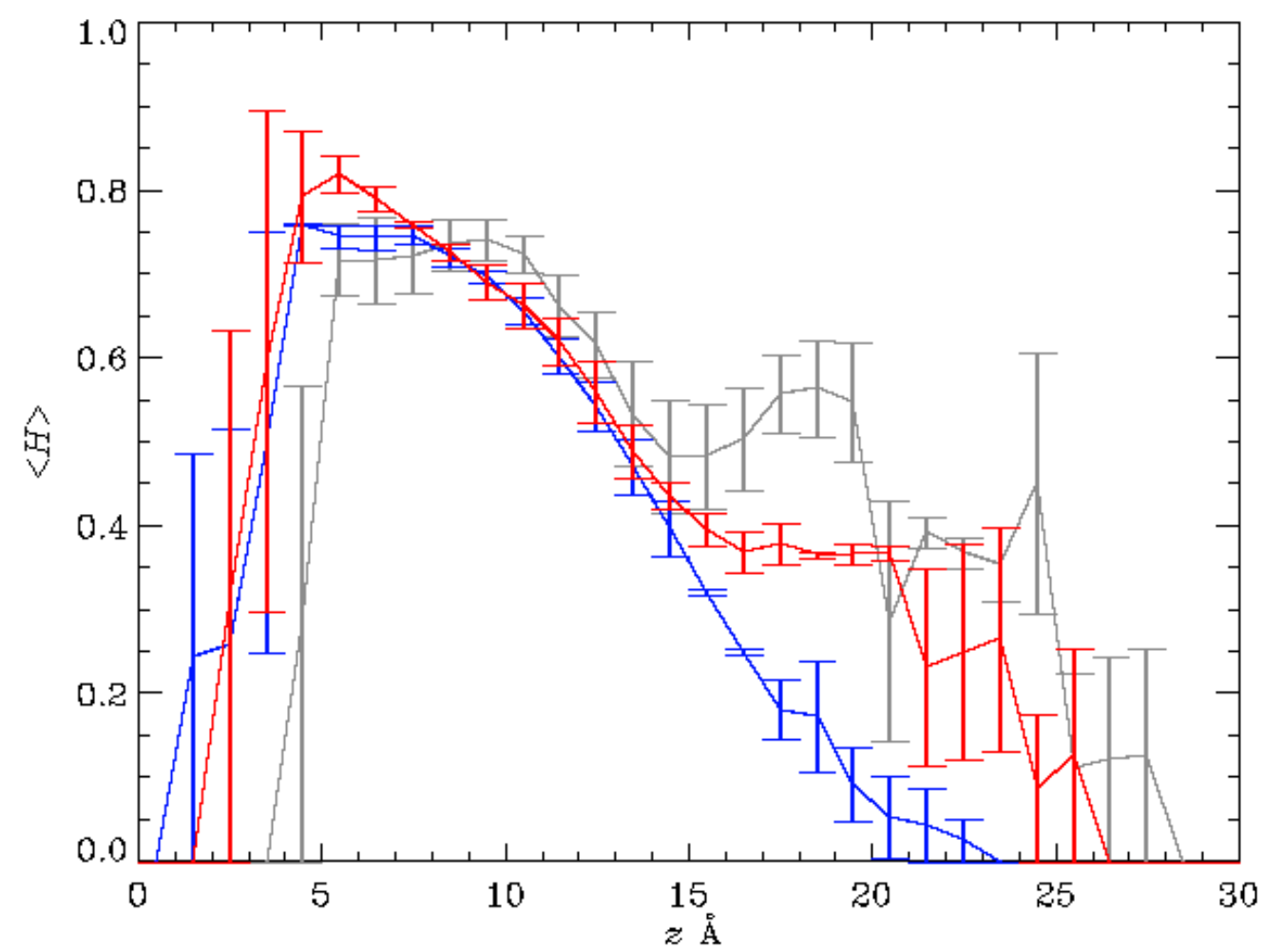

Figure S5 Helical propensity of PGLa <*H*> as a function of the position of its center of mass z_{CM} along the bilayer normal. The data in red, blue, and grey represent HMR3, HMR2, and SM models.

PGLa in lipid-free water: To evaluate the conformational ensemble of PGLa monomer in water, we performed replica exchange with solute tempering simulations using CHARMM36 force field [4,8]. This force field was selected because it produces unstructured peptide conformations in water consistent with experimental data [9]. These similations used *R*= 6 temperature conditions distributed from 310K to 410K. In all, we produced 11 trajectories collecting 1.32 μs of sampling across all conditions and trajectories. These simulations were used to compute the hydration vector <$N_w(i)$> shown in Fig. 6b.

Surface bound state of PGLa is model independent: To verify that the surface bound state **SB** occurs in all models, SM or HMR, we plot the probability distributions $P(z_{CM,Ct})$ of the position of PGLa C-terminus center of mass $z_{CM,Ct}$ along the bilayer normal. The distributions shown in Fig. S6 are collected using 100 ns batches along the REHT timeline in SM and HMR simulations. Table S2 gathers the probabilties of **SB** *P(SB)* in SM and HMR simulations extracted from $P(z_{CM,Ct})$. The results are analyzed in the main text.

(a)

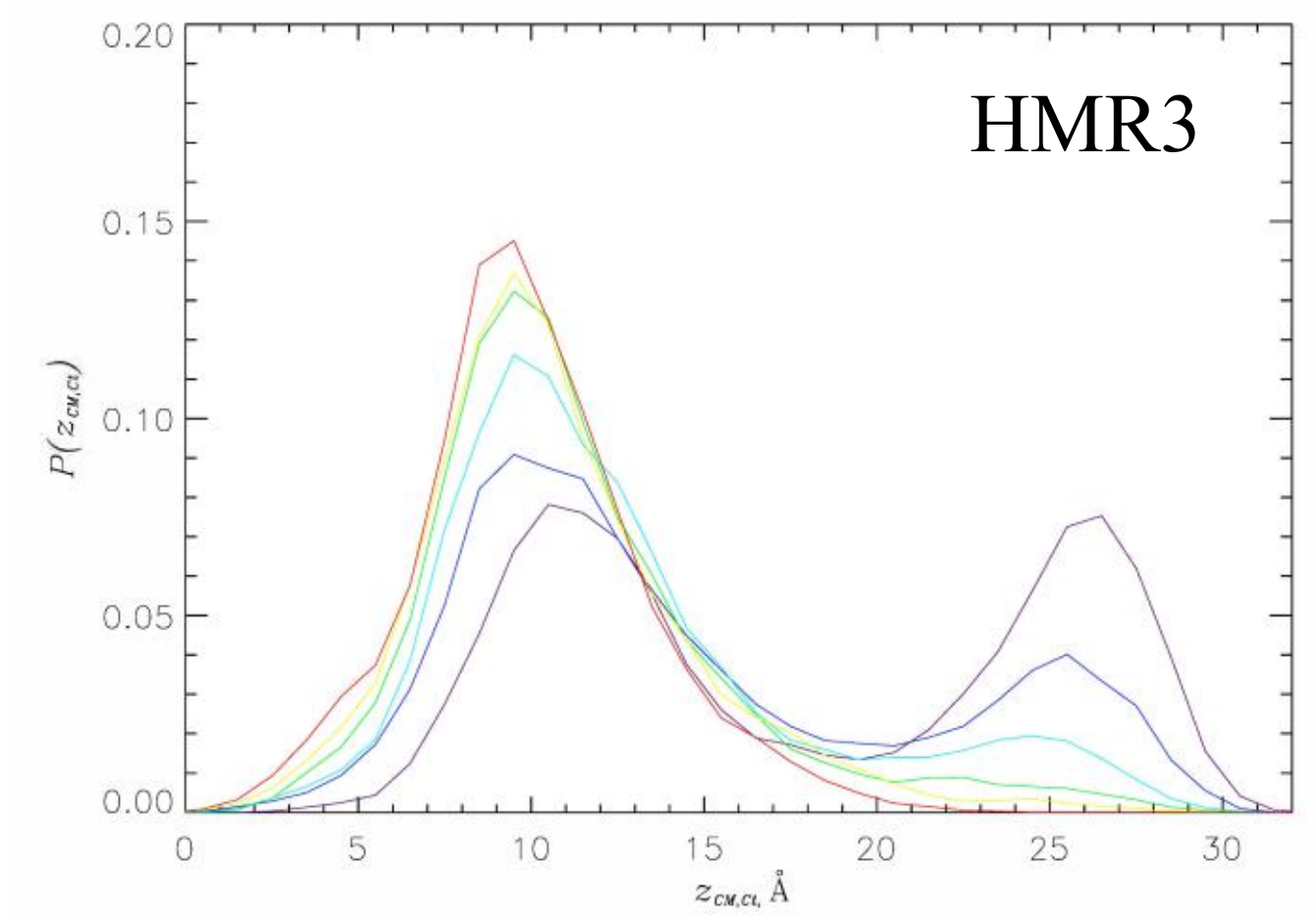

(b)

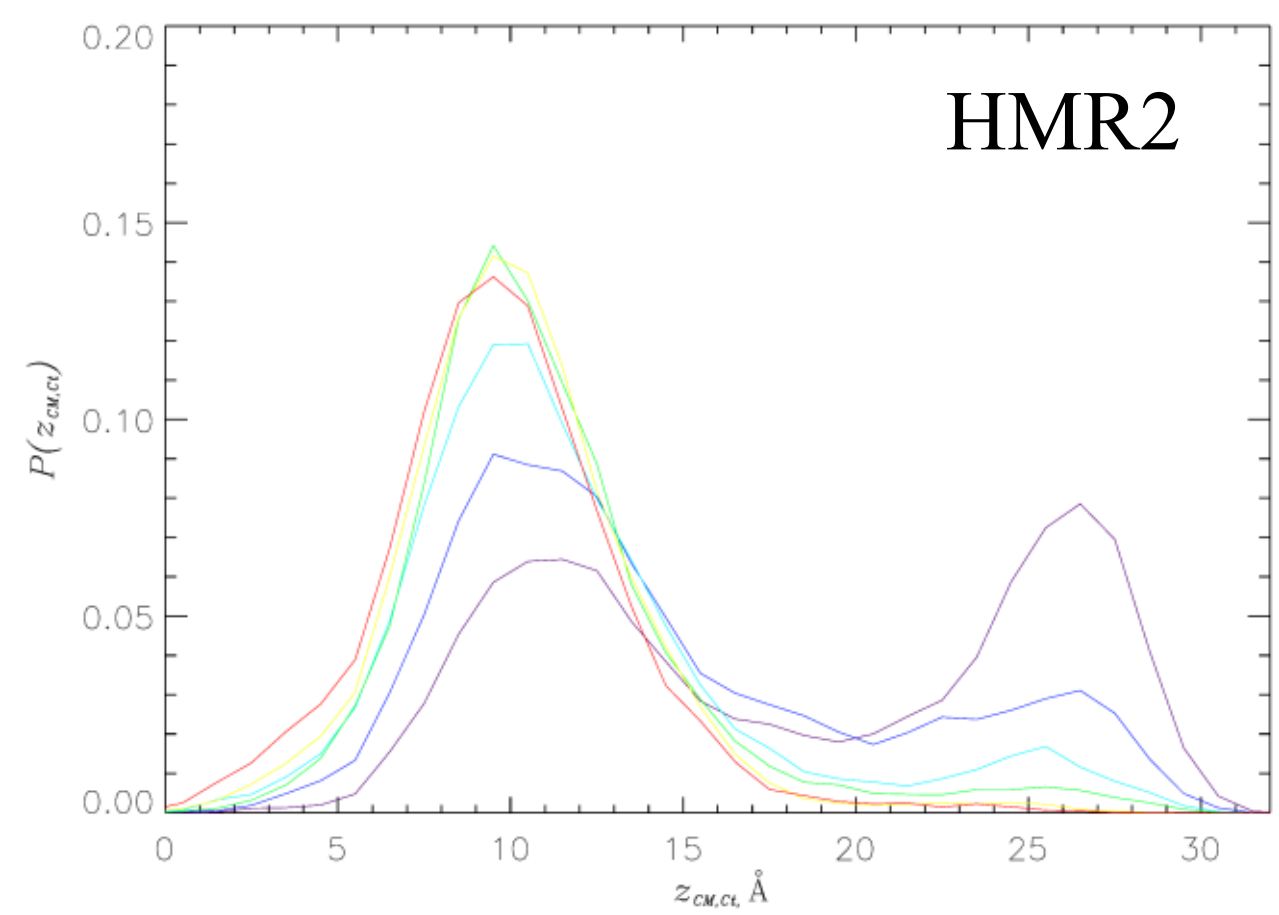

(c)

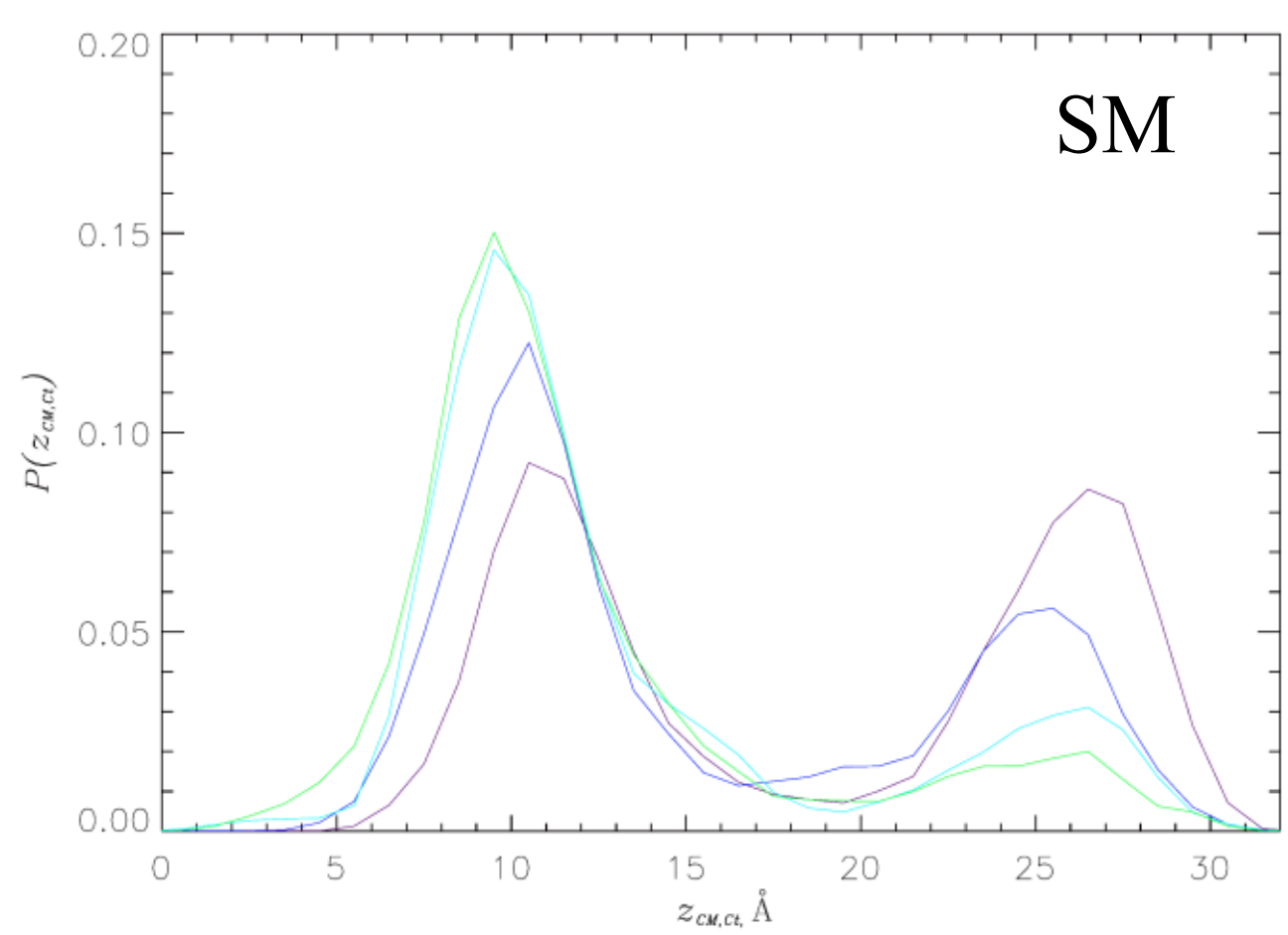

Figure S6 The probability distributions $P(z_{CM,Ct})$ of the position of PGLa C-terminus center of mass $z_{CM,Ct}$ along the bilayer normal. Panels (a) to (c) present the data for HMR3, HMR2, and SM models. The colors distinguish 100 ns batches along the REHT timeline. Because there are three REHT trajectories per model, each batch collects 300 ns of data. The color codes for the batches are purple (ending at 100 ns), blue (200 ns), aqua (300 ns), green (400 ns), yellow (500 ns), red (600 ns).

Table S2 Probabilties *P(SB)* of **SB** state in SM and HMR simulations at different times [a,b]

time batch endpoint, ns	HMR3	HMR2	SM
**100**	0.50	0.54	0.53
**200**	0.33	0.32	0.38
**300**	0.20	0.15	0.22
**400**	0.12	0.09	0.17
**500**	0.10	0.04	
**600**	0.05	0.04	

[a] Probabilities *P(SB)* are computed using $P(z_{CM,Ct})$ distributions and assuming that **SB** occurs at $z_{CM,Ct} > z_0$=16 Å.
[b] *P(SB)* may slighly differ from 1-*P(I)* reported in the main text, because **SB** is defined from $P(z_{CM,Ct})$ distributions, whereas **I** is computed using the free energy landscapes $G(z_{CM,Ct},\beta)$ in Fig. 8.

References:

[1] Bowers, S., Klimov, D. K., Lockhart, C. (2022) Mechanisms of binding of antimicrobial peptide PGLa to DMPC/DMPG membrane *J. Chem. Inf. Model.* doi.org/10.1021/acs.jcim.1c01518.
[2] Bowers, S. R., Lockhart, C., and Klimov, D. K. (2023) Replica Exchange with Hybrid Tempering Efficiently Samples PGLa Peptide Binding to Anionic Bilayer. *J. Chem. Theor. Comput.* **19**, 6532–6550.
[3] Bechinger, B., Zasloff, M., & Opella, S. J. (1998) Structure and Dynamics of the Antibiotic Peptide PGLa in Membranes by Solution and Solid-State Nuclear Magnetic Resonance Spectroscopy. *Biophys. J.* **74**, 981–987.
[4] Huang, J., Rauscher, S., Nawrocki, G., Ran, T., Feig, M., de Groot, B. L., Grubmüller, H., & MacKerell, Jr., A. D. (2016) CHARMM36m: An Improved Force Field for Folded and Intrinsically Disordered Proteins. *Nat. Methods* **14**, 71-73.
[5] Han, M. and Hansmann, U. H. E. (2011) Replica exchange molecular dynamics of the thermodynamics of fibril growth of Alzheimer's Aβ42 peptide. *J. Chem. Phys.* **135**, 065101.
[6] Nadler, W. and Hansmann, U. H. E. (2008) Optimized Explicit-Solvent Replica Exchange Molecular Dynamics from Scratch. *J. Phys. Chem. B* **112**, 10386–10387.
[7] Denschlag, R., Lingenheil, M., and Tavan, P. (2009) Optimal temperature ladders in replica exchange simulations. *Chem. Phys. Lett.* **473**, 193-195.
[8] Wang, L., Friesner, R. A., and Berne, B. J. (2011) Replica Exchange with Solute Scaling: A More Efficient Version of Replica Exchange with Solute Tempering (REST2). *J. Phys. Chem. B* **115**, 9431-9438.
[9] Latal, A., Degovics, G., Epand, R. F., Epand, R. M., Lohner, K. (1997) Structural Aspects of the Interaction of peptidyl-glycylleucine-carboxyamide, a highly potent antimicrobial peptide from frog skin, with lipids. *Eur. J. Biochem*. **248**, 938-946.
